# Copper/iron-based intelligent nanoparticles self-amplify apoptosis/ferroptosis/cuproptosis in colorectal cancer

**DOI:** 10.1016/j.mtbio.2025.102732

**Published:** 2025-12-23

**Authors:** Xuna Xue, Zhibo Zhang, Qiyu Zhang, Xuerong Zhao, Lina Xu, Lianhong Yin, Jinyong Peng, Ning Wang

**Affiliations:** a College of Pharmacy, Dalian Medical University, Western 9 Lvshunnan Road, Dalian, 116044, China; b College of Pharmacy, Hubei Shizhen Laboratory, Hubei University of Chinese Medicine, Wuhan, 430065, China

**Keywords:** Colorectal cancer, Nanoparticle, Multi-pathway synergistic killing, GSH depletion

## Abstract

To address the key challenge of insufficient chemosensitivity in colorectal cancer treatment due to apoptosis resistance, this study proposes a “multi-pathway synergistic killing” strategy that enhances anti-tumour efficacy by simultaneously activating multiple cell death pathways, including apoptosis, ferroptosis, and cuproptosis. Utilising a cRGD targeting peptide, ferrocenecarboxylic acid (Fc), camptothecin (CPT), and doxorubicin (DOX), four amphiphilic molecules were designed and synthesised, which were further assembled and chelated with copper ions to construct multifunctional nanoparticles, cRDT@FC. These nanoparticles can efficiently accumulate in tumour regions. The disulphide bonds and Cu^2+^ within them jointly consume the high concentration of glutathione (GSH) in cells, leading to the disintegration of the nanostructure and the release of CPT, thereby achieving the desired chemotherapy effects. Meanwhile, Cu^2+^ is reduced by GSH to Cu^+^, which, in synergy with Fe^2+^ in the system, catalyses the Fenton reaction to generate a large amount of hydroxyl radicals (·OH) achieving chemodynamic therapy (CDT), significantly enhancing the oxidative stress level and thereby synergistically inducing ferroptosis and cuproptosis. This study presents a novel approach to developing multi-mode cell death synergistic treatment strategies utilising nanotechnology.

## Highlights

• CRDT@FC effectively kills colorectal cancer cells.
• CRDT@FC induces apoptosis, ferroptosis and cuproptosis in cancer cells.
• CRDT@FC is a potential new modality for colorectal cancer treatment.

## Graphical abstract

**Figure:**
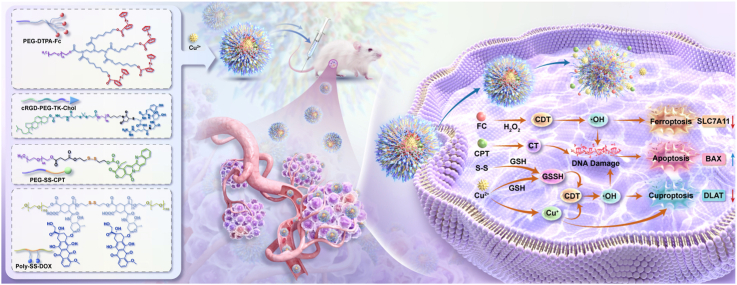

## 1 Introduction

Colorectal cancer is a malignant tumour with high incidence and mortality rates worldwide, posing a significant threat to human health [1]. Despite remarkable progress in early diagnosis and surgical treatment, the prognosis for patients with advanced or metastatic colorectal cancer remains poor. Finding efficient treatment strategies is a significant challenge currently facing researchers. Presently, chemotherapy plays a central role in the comprehensive treatment of colorectal cancer, particularly for patients with advanced and inoperable cases [2]. However, traditional chemotherapy drugs suffer from issues such as poor selectivity, significant toxic side effects, and susceptibility to drug resistance, which greatly limit their clinical efficacy [[3], [4], [5]]. These drugs primarily exert anti-tumour effects by inducing apoptosis in tumour cells [6]. However, apoptosis is a highly regulated cell death pathway, and tumour cells often develop resistance to apoptosis by upregulating anti-apoptotic proteins, leading to chemotherapy failure. Therefore, it is imperative to explore and utilise alternative cell death mechanisms in conjunction with apoptosis to overcome drug resistance and enhance treatment efficacy.

Ferroptosis is a recently discovered type of iron-dependent, regulated cell death that is triggered by the accumulation of lipid peroxides [[7], [8], [9]]. Unlike apoptosis, ferroptosis does not rely on the activation of caspases [10], and its morphological and biochemical characteristics are significantly different from those of apoptosis [11]. Consequently, for tumour cells that have developed resistance to apoptosis, inducing ferroptosis represents a highly promising alternative or synergistic pathway for cell death [[12], [13], [14], [15]]. Following ferroptosis, cuproptosis, a novel copper-ion-dependent cell death mechanism, has been revealed. This mechanism is driven by the direct binding of excessive copper ions to lipid-acylated proteins in the tricarboxylic acid cycle, leading to oligomerisation and ultimately resulting in protein toxicity and cell death [[16], [17], [18]]. Similar to ferroptosis, cuproptosis is not dependent on classical apoptotic pathways, offering new possibilities for targeting and eliminating tumour cells that evade apoptosis [[19], [20], [21], [22]]. In summary, relying solely on the apoptotic pathway presents limitations [23]. Suppose the three distinct and complementary cell death pathways (apoptosis, ferroptosis, and cuproptosis) can be simultaneously activated to create a “multi-pathway synergistic killing” effect. In that case, it is anticipated that this approach will maximise the elimination of tumour cells, thus holding significant promise for improving colorectal cancer treatment [[24], [25], [26], [27], [28]].

Current research on cuproptosis is in its nascent stages, with several critical issues requiring attention. Firstly, the tumour microenvironment typically exhibits relatively low copper levels, which may be insufficient to induce cuproptosis effectively [[29], [30], [31]]. To address this challenge, it is essential to supplement the diet with adequate copper via an exogenous copper delivery system. Although copper ion carriers such as disulfiram, pyrithione, and elesclomol have been developed to enhance cuproptosis [32,33], these small-molecule drugs often encounter limitations, including short circulation times, poor targeting, and rapid clearance at the tumour site, which hinder their efficacy and application [34]. Furthermore, both ferroptosis and cuproptosis are influenced by the robust antioxidant system within cells, particularly the GSH present [[35], [36], [37]]. GSH can effectively reduce lipid peroxides, thereby diminishing ferroptosis driven by lipid peroxide accumulation [38,39]. Excessive GSH may also impact sensitivity to cuproptosis [40]. Based on the above analysis, designing and synthesising a large molecule copper ion chelator that cannot only efficiently chelate copper but also combine with amphiphilic molecules containing iron ions and chemotherapy prodrugs targeting colorectal cancer was proposed, thereby constructing an intelligent nanoparticle that integrates multiple functions [[41], [42], [43], [44], [45]]. This nanoparticle aims to achieve tumour-targeted delivery while consuming GSH within tumour cells, thereby synergistically triggering apoptosis, ferroptosis, and cuproptosis of tumour cells, with the expectation of significantly enhancing the anti-colorectal cancer efficacy.

**Scheme 1:**
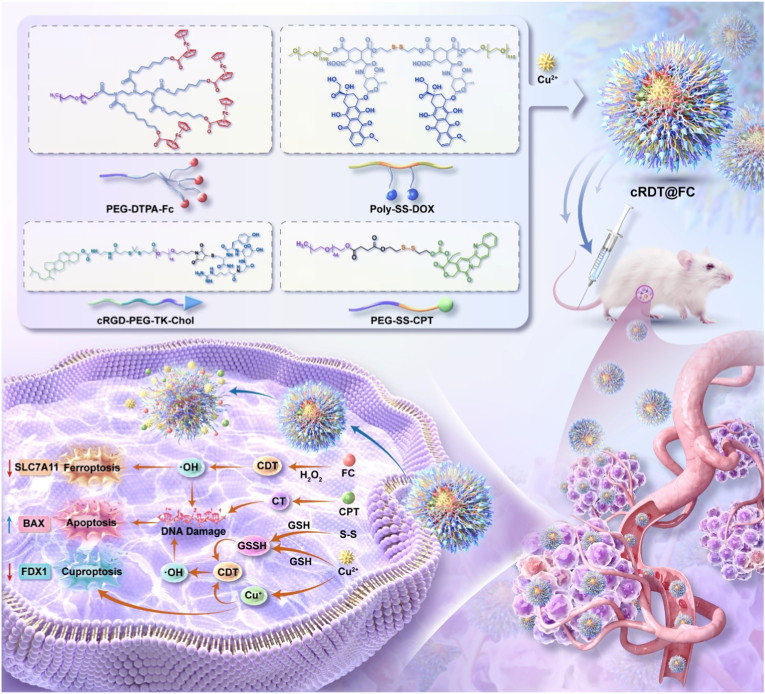
Structural composition and anti-colorectal cancer mechanism of cRDT@FC. The cRDT@FC effectively accumulate at tumour sites, synergistically inducing Apoptosis, Ferroptosis, and Cuproptosis in colorectal cancer.

In this study, an amphiphilic molecule, cRGD-PEG-TK-Chol, was designed to target colorectal cancer cells by covalently linking the circular RGD (cRGD) peptide to the integrin αvβ3 through a Mal-PEG-TK-Chol linker. To enhance the solubility of hydrophobic drugs, the ferroptosis inducer Fc and classic treatment for colorectal cancer, CPT were incorporated into amphiphilic structural units, PEG-DTPA-Fc and prodrug PEG-SS-CPT. DOX is an antitumour drug that inhibits topoisomerase II. Its anthraquinone structure can chelate with copper ions to form a complex; however, free DOX forms a precipitate when chelated with copper ions. Therefore, we modified DOX to synthesize an amphiphilic molecule, Poly-SS-DOX. The coupling of DOX with the intermediate Poly-SS-COOH occurs via amide bonds, which means that DOX does not release but serves only to deliver copper ions to tumour cells, thereby inducing copper-mediated cell death and reducing the toxicity of DOX. cRGD-PEG-TK-Chol, PEG-DTPA-Fc, PEG-SS-CPT, and Poly-SS-DOX were co-assembled to create the intelligent nanoparticle cRDT@FC (Scheme 1). This nanoparticle efficiently accumulates in tumour cells via passive targeting and cRGD-mediated active targeting. Upon entering the cells, the disulphide bonds in cRDT@FC react with the high levels of intracellular GSH, leading to its breakdown and the release of CPT. CPT induces tumour cell apoptosis by inhibiting topoisomerase I activity, thus achieving low-toxicity chemotherapy [46]. Concurrently, Cu^2+^ is reduced to Cu ^+^ by GSH, and together with Fe^2+^ from PEG-DTPA-Fc, it catalyses the Fenton reaction of hydrogen peroxide (H_2_O_2_) achieving CDT [47,48], generating a significant amount of ·OH. This process markedly enhances intracellular oxidative stress, thereby synergistically triggering cuproptosis and ferroptosis. In summary, cRDT@FC integrates multiple cell death mechanisms, effectively elevating oxidative stress levels within tumour cells while significantly reducing the systemic toxicity of free drugs, demonstrating promising application prospects as an intelligent nanomaterial for colorectal cancer treatment.

## 2 Materials and methods

### 2.1 Cells and animals

CT26 cells, MC38 cells, Huvec cells, and Nrk-49F cells were purchased from Procell Life Science & Technology Co., Ltd. Male Balb/c mice (6–8 weeks, 20–22 g) were purchased from Liaoning Changsheng Biotechnology Pty Ltd. All animal experiments in this study followed the National Institutes of Health guidelines and were authorised by the Animal Ethics Committee of Dalian Medical University (Ethics number: XL250816306).

### 2.2 Preparation of nanoparticles

cRDT@FC was synthesised using the thin-film hydration method (Table S1). Initially, cRGD-PEG-TK-Chol, Poly-SS-DOX, PEG-SS-CPT, and PEG-DTPA-Fc were combined in a mass ratio of 1:2.2:2:4 and dissolved in chloroform. The resulting mixture was evaporated under reduced pressure to create a uniform film, which was subsequently dried in a vacuum. The dried film was then hydrated in saline for 10 min. A predetermined amount of CuCl_2_ solution was then added, and the mixture was stirred for 30 min. Unbound Cu^2+^ ions were removed by dialysis (Mw = 3500 Da) against saline. Untargeted nanoparticles (DT@FC) were prepared using Poly-SS-DOX, PEG-SS-CPT, PEG-DTPA-Fc and CuCl_2_ in a mass ratio of 2.2:2:4:4. Targeted nanoparticles without PEG-DTPA-Fc and Cu^2+^ (cRDT) were synthesised using cRGD-PEG-TK-Chol, Poly-SS-DOX, and PEG-SS-CPT in a mass ratio of 1:2.2:2. Targeted nanoparticles loaded with DIR (cRDT@DIR) were prepared with cRGD-PEG-TK-Chol, Poly-SS-DOX, PEG-SS-CPT, and DIR in a mass ratio of 1:2.2:2:1. Similarly, untargeted nanoparticles loaded with DIR (DT@DIR) were synthesised using Poly-SS-DOX, PEG-SS-CPT, and DIR in a mass ratio of 2.2:2:1.

### 2.3 Statistical analysis

All values were expressed as mean ± standard deviation (SD). One-way analysis of variance (ANOVA) was used to compare group means and identify statistically significant differences between them. Statistical significance was set at *p* < 0.05 (*p < 0.05, **p < 0.01).

Detailed descriptions of the remaining methodologies can be found in the supplementary information.

## 3 Results and discussion

### 3.1 cRDT@FC preparation and characteristics

**Fig. 1:**
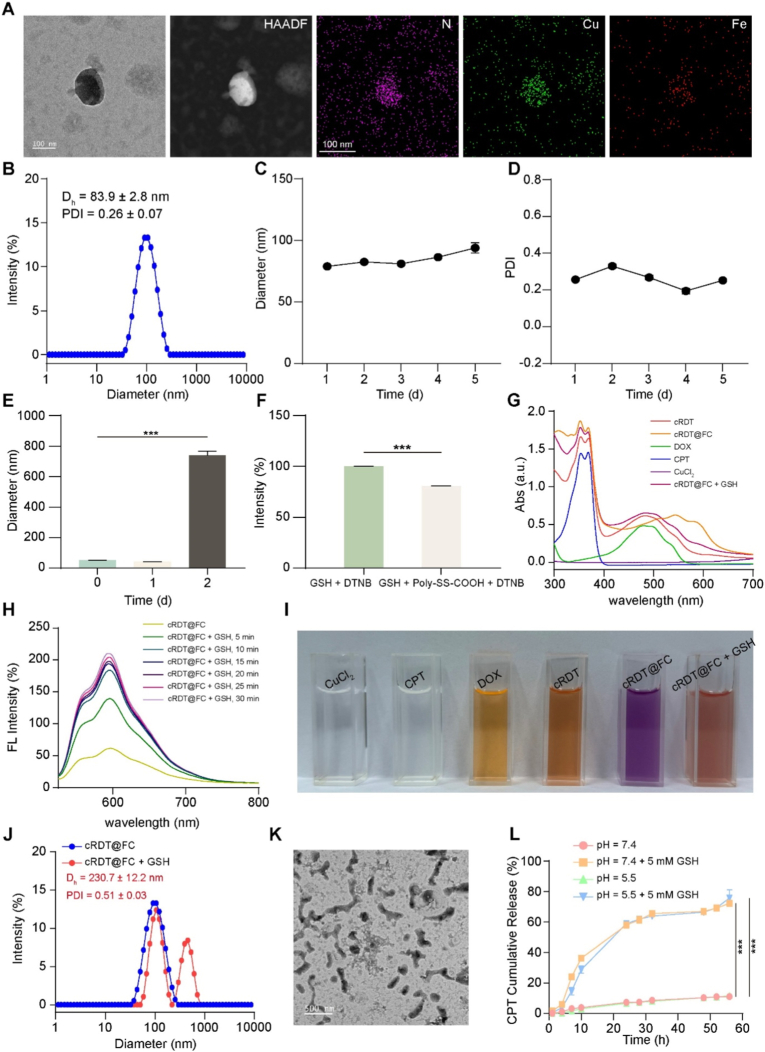
(A) TEM and elemental mapping diagram of cRDT@FC. (B) The particle size distribution of cRDT@FC. Particle size (C) and PDI (D) of cRDT@FC within 5 days. (E) Diameter of cRDT@FC in 10 % FBS conditions with 2 days. (F) Relative intensity of UV absorption of GSH + DTNB and GSH + Poly-SS-COOH + DTNB. (G) UV–Vis absorption spectra of cRDT, cRDT@FC, DOX, CPT, CuCl_2_ and cRDT@FC + GSH. (H) Fluorescence absorption spectra of cRDT@FC at different time points under 5 mM GSH conditions. (I) Concrete figure of cRDT, cRDT@FC, DOX, CPT, CuCl_2_ and cRDT@FC + GSH. (J) The particle size distribution of cRDT@FC and cRDT@FC + GSH. (K) TEM images of cRDT@FC under 5 mM GSH conditions. (L) Cumulative release profile of CPT. ***p < 0.001.

To construct an intelligent nanoparticle with active targeting capability that can synergistically induce various modes of cell death, including apoptosis, ferroptosis, and cuproptosis, four amphiphilic molecules were initially designed and synthesised: cRGD-PEG-TK-Chol, PEG-DTPA-Fc, PEG-SS-CPT, and Poly-SS-DOX **(**Figs. S1–S4**)**. The ^1^H NMR analysis confirmed the successful synthesis of all molecules **(**Figs. S5–S9**)**. UV–Vis absorption spectroscopy revealed that the actual drug loading amounts of PEG-SS-CPT and Poly-SS-DOX are 12 % and 10.8 %, respectively. Subsequently, these four molecules were assembled to construct the intelligent nanoparticle cRDT@FC. Transmission electron microscopy (TEM) and element distribution mapping results demonstrated that cRDT@FC exhibited a regular spherical shape, with elements such as nitrogen (N), copper (Cu), and iron (Fe) uniformly distributed throughout the particles **(**Fig. 1A and Fig. S10**)**. Inductively coupled plasma (ICP) analysis indicates that the copper and iron ion concentrations in the nanoparticles are 1.17 % ± 0.002 and 1.25 % ± 0.002, respectively. Dynamic light scattering (DLS) analysis revealed that the zeta potential of cRDT@FC was −9.54, the average hydrodynamic diameter of cRDT@FC is 83.9 ± 2.8 nm, with a polydispersity index (PDI) of 0.26 ± 0.07, consistent with the TEM observations, indicating the successful preparation and uniform structure of the nanoparticles **(**Fig. 1B and Fig. S11**)**. Furthermore, monitoring the changes in particle size and PDI of cRDT@FC at various time points using DLS revealed stability over 5 days, demonstrating good storage stability **(**Fig. 1C–D**)**. Additionally, following co-incubation with 10 % fetal bovine serum (FBS) for 24 h, the particle size and PDI of cRDT@FC did not exhibit significant changes, indicating good serum stability **(**Fig. 1E and Fig. S12**)**.

The high intracellular concentration of GSH may diminish the therapeutic effects of ferroptosis and cuproptosis; hence, the nanoparticle's ability to consume GSH is critical. Given the presence of disulphide bonds and Cu^2+^ in the structure of cRDT@FC, the DTNB method was employed to evaluate its GSH consumption ability. To avoid the interference of the ultraviolet absorption of CPT and DOX in PEG-SS-CPT and Poly-SS-DOX, a compound rich in disulphide bonds, Poly-SS-COOH, was selected for simulation testing. The results indicated that Poly-SS-COOH significantly depletes GSH **(**Fig. 1F and Fig. S13**).** Moreover, after incubating Poly-SS-COOH + Cu^2+^ with CT26 cells for 4 h, the intracellular GSH concentration was significantly downregulated **(**Fig. S14**)**. In summary, these results indicate that cRDT@FC possesses a high capacity for GSH consumption, which may enhance the therapeutic effects of ferroptosis and cuproptosis. To assess the responsiveness of cRDT@FC to GSH, a comprehensive evaluation was undertaken employing various techniques, including UV–Vis spectroscopy, fluorescence spectroscopy, DLS and TEM. The data reveal that upon the addition of GSH, the maximum absorption wavelength of cRDT@FC shifts from 544 nm to 483 nm, with a significant enhancement in fluorescence intensity at 594 nm **(**Fig. 1G–I**)**. DLS and TEM analyses show that the particle size of cRDT@FC increased and the PDI rose after treatment with 5 mM GSH, accompanied by a morphological transition from spherical to irregular, thereby illustrating its robust responsiveness to GSH **(**Fig. 1J–K**)**. CPT, recognised as a classic anti-cancer agent for colorectal cancer, induces tumour cell apoptosis by inhibiting topoisomerase I activity. For effective anti-tumour outcomes, cRDT@FC must efficiently release CPT within the tumour microenvironment. The research findings indicate that under the conditions of pH = 7.4 + GSH, cRDT@FC can release approximately 72.3 % of CPT within 56 h, which is 6.51 times that of the control group. Notably, under the conditions of pH = 5.0 + GSH, the cumulative drug release of CPT is close to that at pH = 7.4 + GSH, suggesting that slightly acidic conditions do not affect the drug release of CPT **(**Fig. 1L**)**. This finding further corroborates the exceptional drug-controlled-release capability and GSH consumption potential of cRDT@FC. The copper/iron ions within cRDT@FC can react with H_2_O_2_ in tumour cells through a Fenton-like and Fenton reaction, generating highly reactive ·OH. This characteristic was validated using electron spin resonance (ESR) technology, the gold standard for detecting reactive oxygen species (ROS). cRDT@FC exhibits a distinct ·OH characteristic signal peak in the presence of H_2_O_2_, indicating that this nanoparticle can effectively elevate the ·OH levels within tumour cells, augment oxidative stress, and consequently promote ferroptosis and cuproptosis **(**Fig. S15**)**.

### 3.2 cRDT@FC targeting and anti-tumour activity *in vitro*

**Fig. 2:**
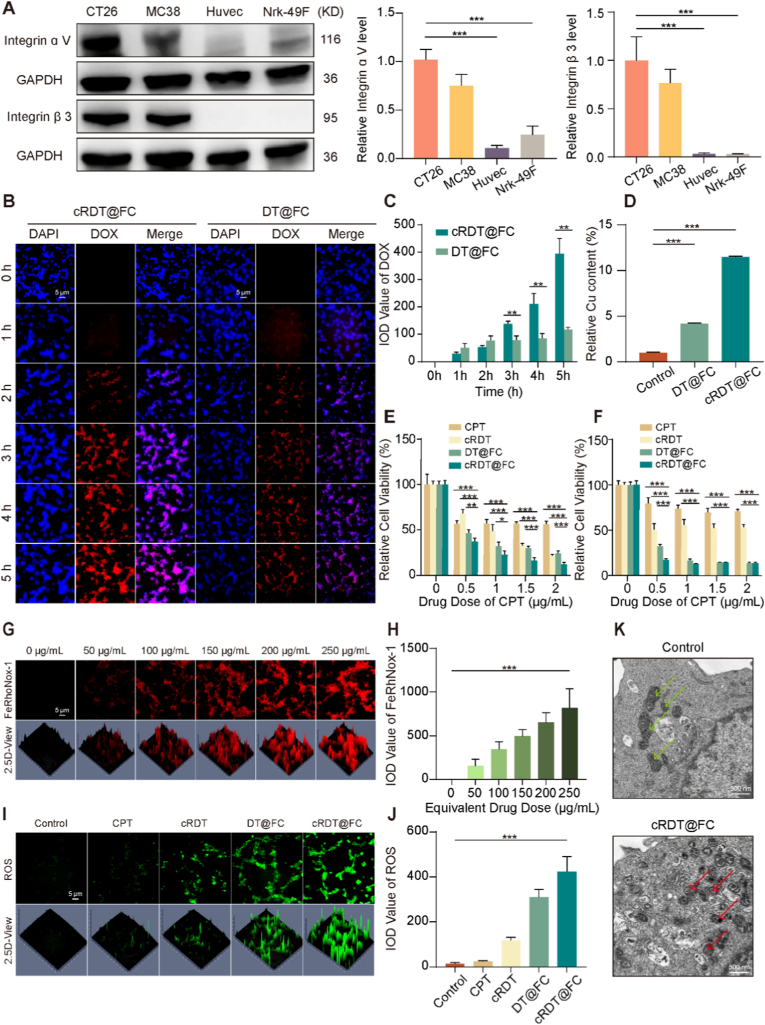
(A) The protein expression of Integrin ɑ Ⅴ and Integrin β 3 in CT26, MC38, Huvec and Nrk-49F cells. (B) Images of cRDT@FC uptake by CT26 cells at different time periods. (C) Fluorescence quantitative images of Fig. 2B. (D) Relative Cu content in CT26 cells of Saline, DT@FC and cRDT@FC group, Data are presented as mean ± SD (n = 3). The cell viability of different preparations to CT26 cells (E) and MC38 cells (F) after 24 h (n = 6). *p < 0.05, **p < 0.01, ***p < 0.001. (G) The fluorescent inverted microscope image of intracellular Fe^2+^ detected using the FeRhoNox-1 kit. (H) Fluorescence quantitative images of Fig. 2G. (I) The fluorescent inverted microscope images of ROS generation in CT26 cells treated with different preparations. (J) Fluorescence quantitative images of Fig. 2I. (K) Bio-TEM images of Control and cRDT@FC incubated with CT26 cells for 24 h, respectively. PBS as a Control. *p < 0.05, **p < 0.01.

Nanoparticle formulations exhibit remarkably active targeting capabilities, which effectively reduce drug toxicity while enhancing anti-tumour efficacy. Building on this characteristic, the present study systematically evaluated the *in vitro* targeting performance of cRDT@FC. The peptide cRGDyc, modified in cRDT@FC, specifically recognises and binds to integrin αvβ3. Therefore, Western Blot technology was used to assess the protein expression levels of αvβ3 in CT26 and MC38 cells. As illustrated in Fig. 2A, the expression levels of αvβ3 in both colorectal cancer cell lines are significantly elevated compared to those in normal cells (Huvecs and Nrk-49F), indicating that cRDT@FC has a promising basis for targeted treatment of colorectal cancer. Subsequently, inverted fluorescence microscopy was utilised to observe the uptake behaviour of CT26 cells with cRDT@FC. The results demonstrated that the DOX fluorescence signal in the cells gradually increases over time, suggesting that cRDT@FC can effectively penetrate tumour cells. Notably, at each time point, the DOX fluorescence intensity in the cRDT@FC group is significantly greater than that in the non-targeted group DT@FC, further confirming its superior targeting capability for colorectal cancer **(**Fig. 2B–C**)**.

To further investigate the active targeting ability of cRDT@FC, cRDT@FC and DT@FC were co-incubated with CT26 cells for 24 h, followed by the detection of intracellular copper ion content using ICP-MS. As illustrated in Fig. 2D, the copper ion content in the cRDT@FC group is significantly higher than that in both the Control group and the DT@FC group. This finding aligns with the results observed through fluorescence microscopy, collectively validating the active targeting performance of cRDT@FC. Following the confirmation of its targeting ability, the *in vitro* anti-tumour effects of cRDT@FC were further evaluated. As depicted in Fig. 2E–F, cRDT@FC demonstrated significant growth inhibition effects on CT26 and MC38 cells. To assess its biocompatibility, cRDT@FC was co-incubated with normal cells, Nrk-49F, for 24 h, after which cell viability was evaluated. The results indicated that at a CPT concentration of 2 μg/mL, the survival rate of Nrk-49F cells is approximately 5.7 times that of CT26 cells, suggesting that cRDT@FC exhibits low toxicity towards normal cells and possesses good biological safety **(**Fig. S16**)**. During verification of targeting ability, it was noted that the copper ion concentration in the cRDT@FC group is approximately 11.4 times greater than that in the Control group, implying that it may induce cuproptosis by promoting intracellular copper ion accumulation **(**Fig. 2D**)**.

To investigate whether cRDT@FC influences ferroptosis, the FeRhoNOX-1 probe was employed to measure the intracellular Fe^2+^ levels in cells. Due to fluorescence interference from DOX, a different concentration of PEG-DTPA-Fc was utilised for incubation with CT26 cells. The results indicated that the red fluorescence increased with higher concentrations **(**Fig. 2G–H**)**, suggesting that cRDT@FC could elevate the Fe^2+^ levels in tumour cells, thereby creating conditions conducive to ferroptosis induction. The accumulation of copper and iron ions within cells can engage in Fenton-like or Fenton reactions with hydrogen peroxide, leading to the generation of ROS. Employing a ROS detection kit revealed that both the DT@FC and cRDT@FC treatment groups exhibited pronounced green fluorescence signals, indicating that both treatments could trigger ROS bursts **(**Fig. 2I–J**)**. Notably, the fluorescence intensity in the cRDT@FC group is 1.4 times greater than that in the DT@FC group, further confirming its superior targeting efficacy. Ferroptosis and cuproptosis are often accompanied by damage to mitochondrial morphology. To elucidate the mechanism of action of cRDT@FC, a biotransmission electron microscope (Bio-TEM) was used to examine the ultrastructure of mitochondria in CT26 cells. The results demonstrated that, compared to the Control group, the mitochondrial cristae in cells treated with cRDT@FC are significantly reduced or even absent **(**Fig. 2K**)**. This finding indicates that cRDT@FC can induce ferroptosis and cuproptosis in tumour cells, thereby killing tumour cells that are resistant to apoptosis and enhancing anti-tumour efficacy.

### 3.3 cRDT@FC acute toxicity experiment

**Fig. 3:**
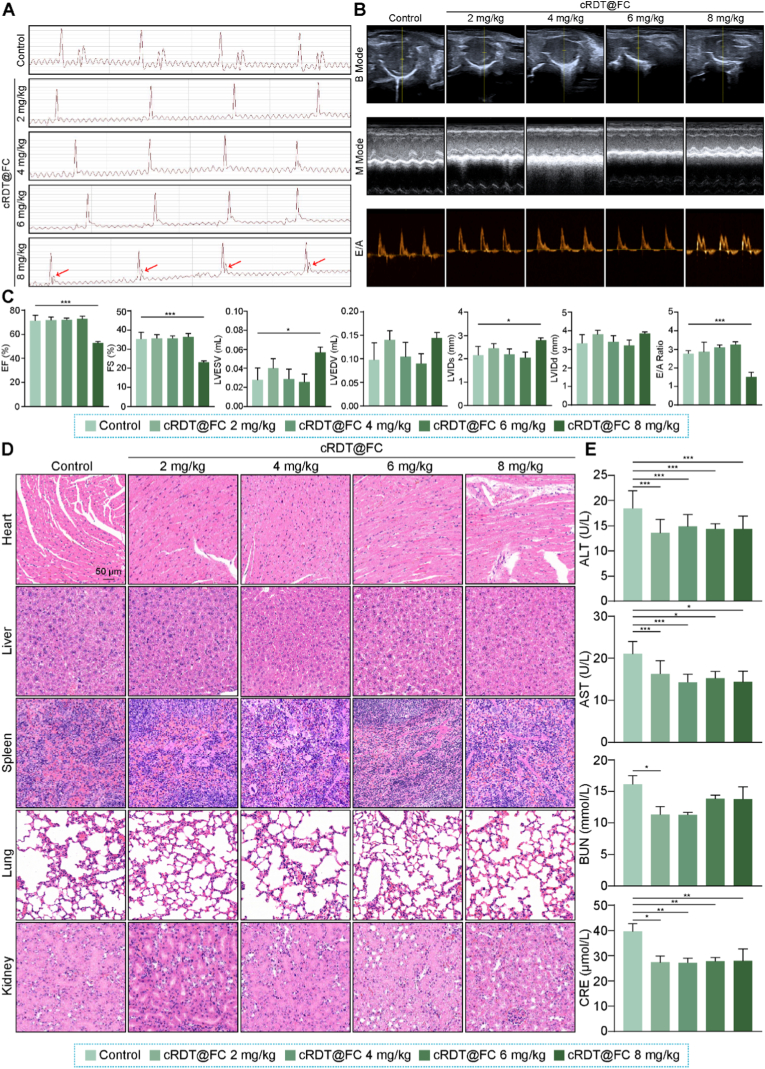
(A) Electrocardiogram, (B) B Mode, M Mode and E/A images of mouse heart after tail vein injection of different concentrations of cRDT@FC for 24 h. (C) The numerical values of EF%, FS%, LVES V, LVED V, LVID s, LVID d and E/A in each group. (D) HE images after tail vein injection of different concentrations of cRDT@FC for 24 h. (E) Biochemical analysis values after tail vein injection of different concentrations of cRDT@FC for 24 h *p < 0.05, **p < 0.01, ***p < 0.001.

Based on the significant anti-tumour activity demonstrated by cRDT@FC *in vitro*, further data were explored to evaluate its anti-tumour effects *in vivo*. However, the suitability of cRDT@FC for intravenous administration and its safe dosing range still requires clarification. The blood compatibility of its constituent materials: RGD-PEG-TK-Chol, PEG-DTPA-Fc, PEG-SS-CPT, and Poly-SS-DOX was initially assessed. The results indicated that the hemolysis rates of all four amphiphilic molecules are below 10 % at a concentration of 400 μg/mL, suggesting good blood compatibility and suitability for intravenous administration **(**Figs. S17–18**)**. Subsequently, varying doses (2, 4, 6, and 8 mg/kg) of cRDT@FC were injected into Balb/c mice via the tail vein, and analyses were conducted 24 h later. Given that cRDT@FC contains DOX, a small animal electrocardiogram instrument and an ultrasound imaging system were used to assess its potential cardiac toxicity. The electrocardiogram analysis revealed that the 8 mg/kg dose group exhibited ST-segment elevation, indicating that this concentration may have toxic effects on the heart **(**Fig. 3A**)**. Further cardiac ultrasound examinations and HE staining results corroborated the electrocardiogram findings, collectively confirming that the 8 mg/kg dose exhibited specific cardiac toxicity in mice **(**Fig. 3B–D**)**. Additionally, the ALT, AST, BUN, and CRE indicators were monitored in each group of mice to evaluate their impact on liver and kidney functions. The results showed that, compared to the Control group, none of the indicators in the administration groups increased **(**Fig. 3E**)**, indicating that cRDT@FC did not cause significant liver or kidney damage within a dose range of up to 8 mg/kg. Based on these findings, 6 mg/kg was used as the administration dose of cRDT@FC in subsequent *in vivo* anti-tumour experiments.

### 3.4 Targeting *in vivo* and anti-tumour experiments

**Fig. 4:**
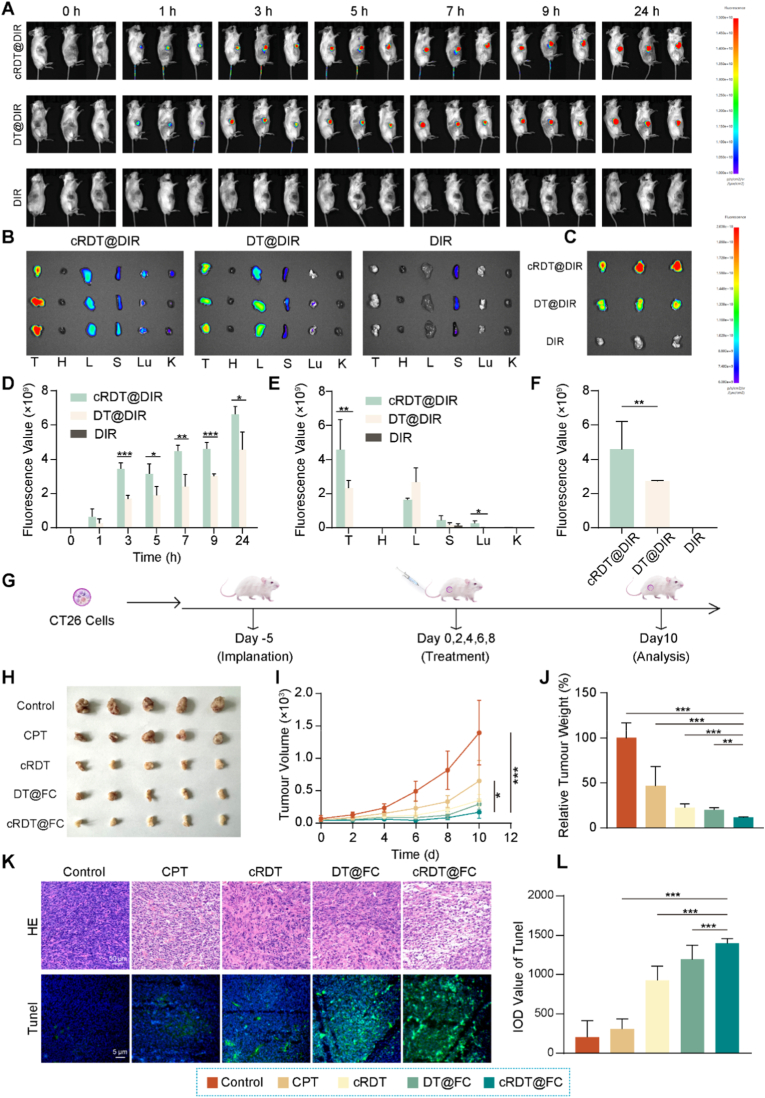
(A) Fluorescent images of tumour-bearing mice at different time points after tail vein injection of fluorescent dye DIR, DT@DIR and cRDT@DIR. (n = 3). Fluorescent images of various organs (B) and tumour (C) in tumour-bearing mice after 24 h of tail vein injection of fluorescent dye DIR, DT@DIR and CRDT@DIR. Fluorescence quantitative images of (D) Fig. 4A–(E) Fig. 4B and (F) Fig. 4C. (G) Construction of CT26 tumour-bearing mice and schematic diagram of drug administration. (H) The tumour comparison for each group at the end of treatment. (I) Tumour volume change curves of mice for every other treatment group during the treatment period. (J) Relative tumour weight of mice for every other treatment group during the treatment period. (K) HE and Tunel staining of tumour in each treatment groups at the end of treatment. (L) Fluorescence quantitative images of Tunel in Fig. 4K (n = 3). *p < 0.05, **p < 0.01, ***p < 0.001.

The effective anti-tumour effect of cRDT@FC relies on its capacity to accumulate in tumour tissues. To validate this critical aspect, a small animal *in vivo* imaging system was used to assess whether cRDT@FC could penetrate the tumour regions of mice. Given that the CPT and DOX molecules within cRDT@FC exhibit relatively limited tissue penetration, the classic *in vivo* near-infrared fluorescent dye DIR was selected as a tracer for this study. Targeted nanoparticles, cRDT@DIR, and non-targeted nanoparticles, DT@DIR, were both developed and constructed. The imaging results demonstrated that both groups of nanoparticle-treated mouse tumour regions displayed significant fluorescence signal enhancement, indicating effective accumulation of cRDT@DIR and DT@DIR at the tumour sites **(**Fig. 4A–F and Fig. S19-20**)**. Notably, the fluorescence intensity in the cRDT@DIR group is significantly greater than in the DT@DIR group, confirming the active targeting capability of cRDT@FC. Furthermore, 24 h post-injection, the fluorescence signal in the tumour centre of the cRDT@DIR group is markedly stronger than that in the peripheral area, suggesting that cRDT@FC can penetrate deeply into the tumour core, which is anticipated to enhance its cytotoxic effect on deep tumour cells.

To further evaluate the comprehensive efficacy of cRDT@FC-mediated apoptosis, ferroptosis, and cuproptosis in anti-tumour activity, a Balb/c mouse tumour model of CT26 colorectal cancer was established **(**Fig. 4G**)**. When the tumour volume reached approximately 60 mm^3^, the mice were randomly divided into five groups (n = 5): 1) Control group; 2) CPT group; 3) cRDT group; 4) DT@FC group; 5) cRDT@FC group. Each group received treatment every two days for a total of five administrations. The experimental results **(**Fig. 4H–J**)** indicate that, compared to the Control group, the CPT group exhibits only mild inhibition of tumour growth. In contrast, each nanoparticle formulation group demonstrates significant anti-tumour effects, primarily due to the passive targeting accumulation of nanoparticles in tumour tissues via the enhanced permeability and retention (EPR) effect. Notably, cRDT@FC exhibits a powerful anti-tumour capability, with a tumour suppression rate of approximately 88 ± 6.9 %, compared to the apoptosis group cRDT. This demonstrates the advantages of combined multicellular death modalities. Histological analysis reveals that tumour cells in the cRDT@FC group were more loosely arranged, and the tissue structure is more compromised. TUNEL staining results indicate that the green fluorescence signal in the cRDT@FC group is the strongest, further confirming that cRDT@FC could synergistically induce multiple modes of cell death and exert a potent anti-tumour effect **(**Fig. 4K–L**)**.

### 3.5 Biological safety

**Fig. 5:**
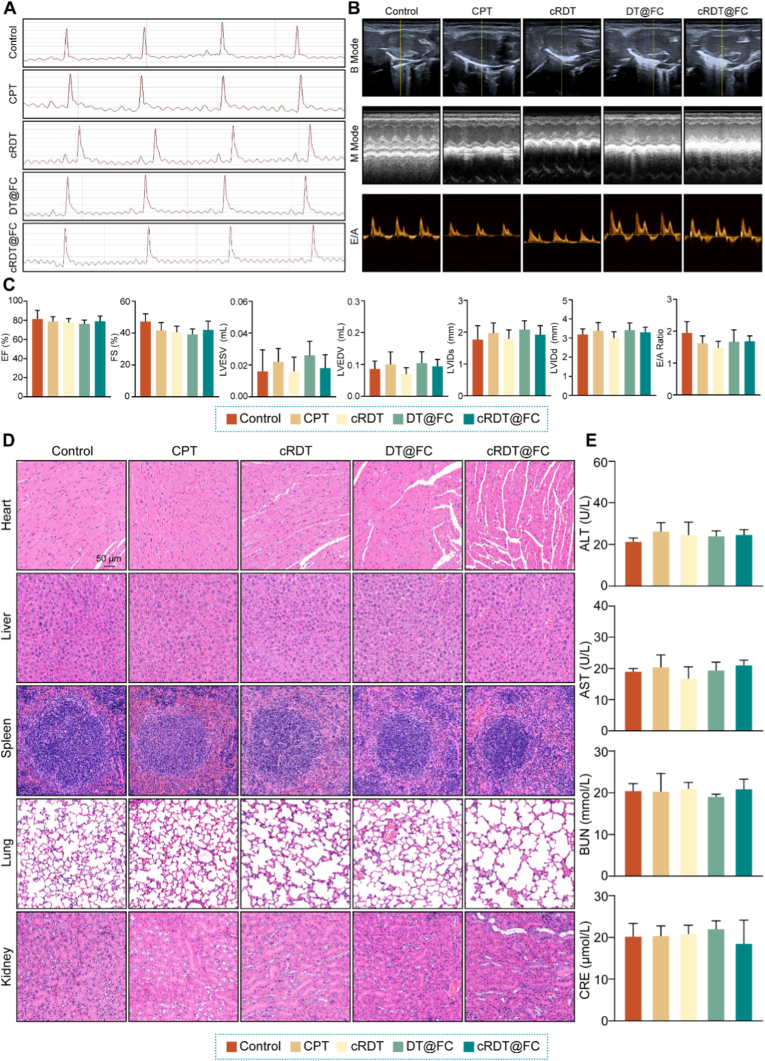
(A) Electrocardiogram, (B) B Mode, M Mode and E/A images of mouse heart in different treatment groups after the *in vivo* anti-tumour experiment. (C) The numerical values of EF%, FS%, LVES V, LVED V, LVID s, LVID d and E/A in each group. (D) HE staining of tumour-bearing mice in different treatment groups. (E) Biochemical analysis values of tumour-bearing mice in different treatment groups.

To ensure the biological safety of the nanoparticles, this study conducted a systematic evaluation of the safety profile of cRDT@FC. Initially, weight changes in each group of mice were recorded before and after administration **(**Fig. S21**)**, revealing no significant fluctuations in weight. This preliminary observation indicated that cRDT@FC exhibits good biocompatibility. Following treatment, a comprehensive safety evaluation was performed using various methods, including small animal electrocardiogram equipment, ultrasound imaging systems, HE staining, and liver and kidney function test kits **(**Fig. 5A–E**)**. The results indicate no significant differences in various physiological indicators between the cRDT@FC group and the Control group, further confirming the appropriate biological safety of cRDT@FC *in vivo*.

### 3.6 Anti-colorectal cancer mechanism of cRDT@FC

**Fig. 6:**
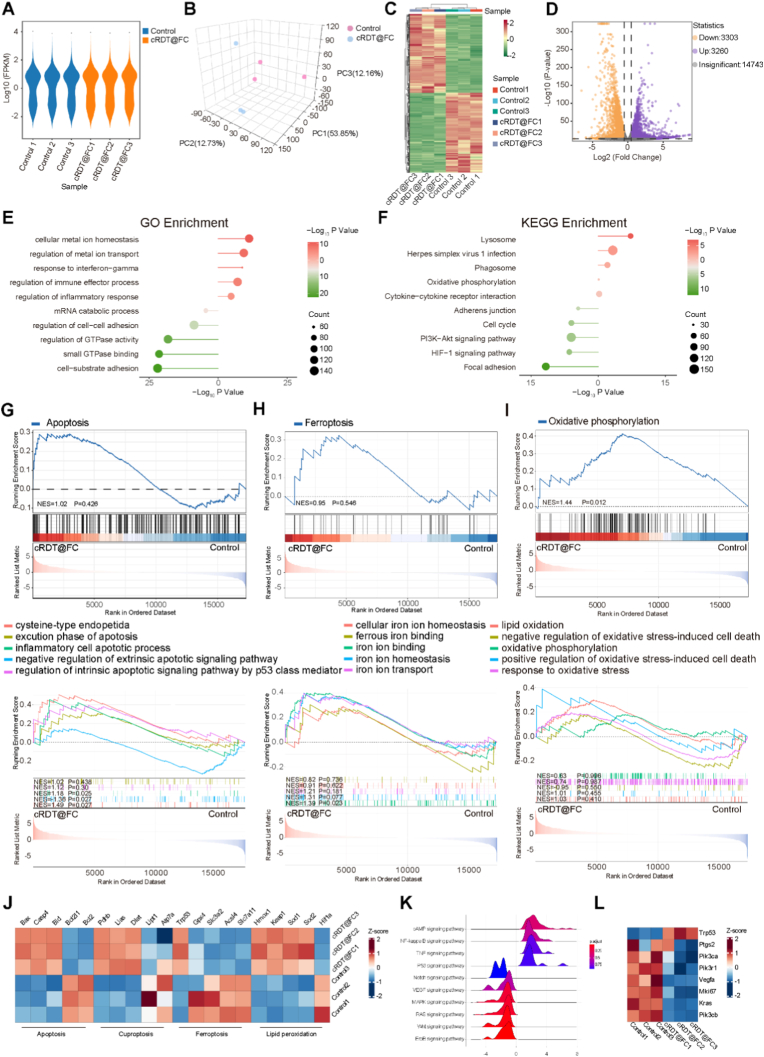
(A) Expression violin plots for the Control group and the cRDT@FC group of RNA-seq. (B) Principal Component Analysis (PCA) of the samples from cRDT@FC group and Control group. (C) Heatmap of the DEGs from cRDT@FC group versus Control group. (D) Volcano plot demonstrated the differential analysis result with the threshold of Log_2_|FC| ≥0.5 and P value < 0.05 of cRDT@FC group versus Control group from RNA-seq. (E) Enrichment analysis of Up and Down genes of DEGs by GO pathway. (F) Enrichment analysis of Up and Down genes of DEGs by KEGG pathway. (G) GSEA of Apoptosis related pathways in cRDT@FC treated CT26 cells versus Control. (H) GSEA of Ferroptosis related pathways in cRDT@FC treated CT26 cells versus Control. (I) GSEA of Lipid peroxidation related pathways in cRDT@FC treated CT26 cells versus Control. (J) Heatmap of gene expression related to Apoptosis, Cuproptosis, Ferroptosis and Lipid peroxidation in CT26 cells treated with CRDT@FC versus Control. (K) Ridgeplot of pathway enrichment analysis diagram in cRDT@FC treated CT26 cells versus Control. (L) Heatmap of expression of indicators related to clinical detection of colorectal cancer in CT26 cells with cRDT@FC versus Control.

The key to enhancing the anti-tumour efficacy of cRDT@FC lies in inducing apoptosis, ferroptosis, and cuproptosis in tumour cells. Based on this, the molecular mechanisms of cRDT@FC against colorectal cancer were explored using techniques such as RNA sequencing (RNA-seq), Western blot, immunofluorescence, and Bio-TEM. Firstly, RNA was collected and extracted from PBS and cRDT@FC-treated CT26 cells, respectively, and RNA sequencing analysis was performed. Gene expression distribution, Principal Component Analysis (PCA), and sample correlation results **(**Fig. 6A–B and Fig. S22**)** show that the cRDT@FC group have a similar gene expression distribution to the Control group. The distribution of samples within the groups is homogeneous, and there is a clear clustering relationship between the distributions of the groups. As the heatmap results show **(**Fig. 6C**)**, there is a significant change in gene expression after treating cells with cRDT@FC compared to the Control group. Based on the above, the analysis of Differentially Expressed Genes (DEGs) was conducted under the condition of log_2_|FC| ≥ 0.5 and P < 0.05. The volcano plot results show **(**Fig. 6D**)** a total of 6563 DEGs after administration of cRDT@FC, of which 3303 are down-regulated and 3260 are upregulated. Subsequently, Gene Ontology (GO) enrichment analysis and Kyoto Encyclopedia of Genes and Genomes (KEGG) enrichment analysis were performed on the upregulated and down-regulated DEGs **(**Fig. 6E–F and Fig. S23-24**)** As the results show, after cellular administration of cRDT@FC, the functions of “regulation of metal ion transport”, “regulation of inflammatory response” and “oxidative phosphorylation” are significantly upregulated. In contrast, the pathways of cell adhesion, GTPase and cell cycle are significantly down-regulated. It was further demonstrated that the mechanism of cRDT@FC against colorectal cancer is partly realised through apoptosis and imbalance of metal ion homeostasis. To better illustrate the mechanism of cRDT@FC against colorectal cancer, the data were further analysed using Gene Set Enrichment Analysis (GSEA). The results of GSEA-KEGG and GSEA-GO analyses show that the Apoptotic pathway and the function of “inflammatory cells apoptotic process” are significantly upregulated **(**Fig. 6G**)**, the Sulphur metabolism pathway and its function are significantly different **(**Fig. S25**)**, the Ferroptosis pathway and the function of “iron ion binding” are significantly upregulated **(**Fig. 6H**)** and the oxidative phosphorylation pathway and the function of “lipid oxidation” are significantly upregulated **(**Fig. 6I**)**. Further, the heatmap of gene expression related to the above four biological processes **(**Fig. 6J**)**, shows that the pro-apoptotic gene BAX is upregulated, the gene promoting cuproptosis process DLAT is upregulated, the ferroptosis resistance gene SLC7A11 is down-regulated and the lipid peroxidation-related gene Keap1 is upregulated in the cRDT@FC group compared to the Control group. Such data are consistent with the above results and demonstrate that cRDT@FC inhibits colorectal cancer by simultaneously inducing apoptosis, ferroptosis, and cuproptosis. As shown in the GSEA pathway enrichment analysis **(**Fig. 6K**)**, the NF-kappa B, TNF, and P53 signaling pathways were significantly upregulated after drug administration. In contrast, pro-cancer pathways, such as RAS and Wnt, are significantly downregulated, further confirming that cRDT@FC is effectively inhibiting tumour development. Additionally, some clinical prognostic indicators of colorectal cancer were selected for analysis **(**Fig. 6L**)**. The heatmap results show that genes encoding tumour-suppressor proteins such as Trp53 are significantly upregulated in the cRDT@FC group compared to the Control group. In contrast, gene indicators that promote tumourigenesis or are highly expressed in tumours, such as Vegfa, Mki67, and Pik3cb, were significantly downregulated, suggesting that cRDT@FC also favours the prognosis of colorectal cancer.

**Fig. 7:**
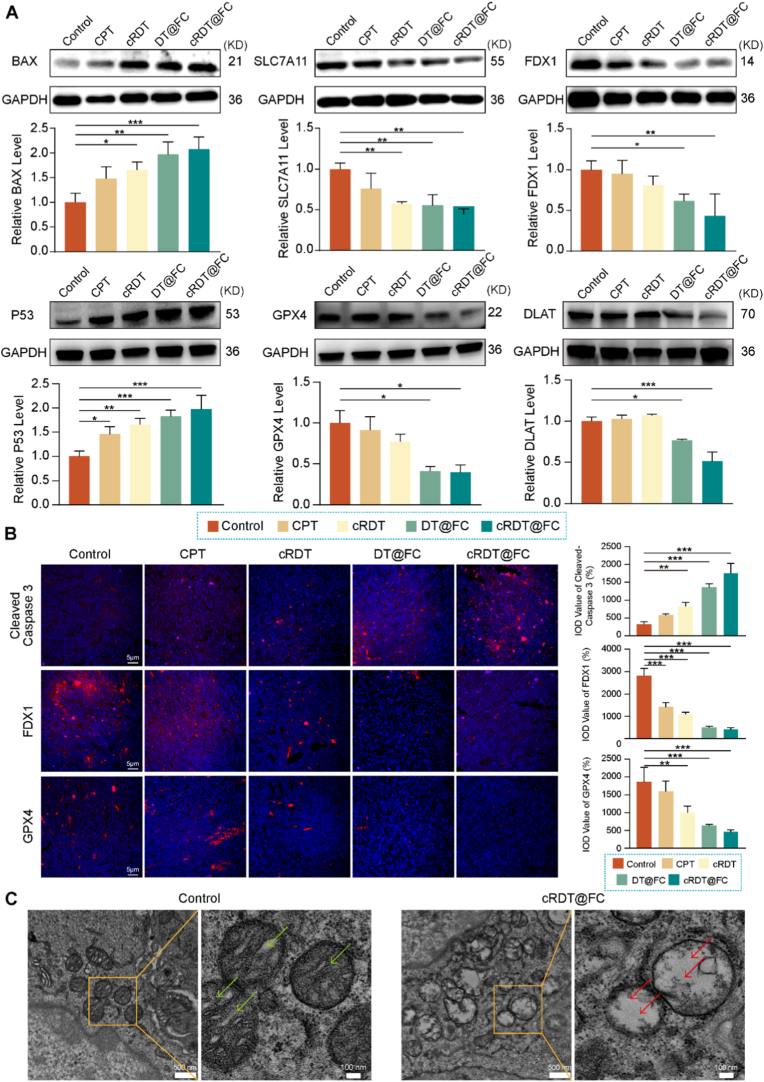
(A) After incubating with various preparations for 24 h, the protein expression of BAX, P53, SLC7A11, GPX4, FDX1 and DLAT (B) Immunofluorescence staining and corresponding fluorescence quantitative images of Cleaved-caspase3, FDX1 and GPX4 of tumours in different treatment groups after the *in vivo* anti-tumour experiment. (C) Bio-TEM images of tumour in the Control and cRDT@FC groups after the *in vivo* anti-tumour experiment. PBS as a Control. *p < 0.05, **p < 0.01, ***p < 0.001.

Based on the above sequencing data, the mechanism of cRDT@FC treating colorectal cancer was also investigated and verified at the protein level **(**Fig. 7A**)**. The data show that, compared with the Control data, cRDT@FC treatment significantly upregulated the pro-apoptotic protein BAX, P53 and downregulated the cuproptosis-associated protein FDX1, DLAT, as well as the ferroptosis-resistant protein SLC7A11 and GPX4. The experimental results align with the sequencing results, further confirming that cRDT@FC induces apoptosis, cuproptosis, and ferroptosis in tumours. Additionally, staining of mouse tumour tissues shows **(**Fig. 7B**)** that Cleaved-Caspase3 red fluorescence intensity is the highest in the cRDT@FC group, and the red fluorescence intensities of FDX1 and GPX4 are the lowest in the cRDT@FC group compared with the Control group. Such outcomes support the hypothesis that cRDT@FC exerts its anti-colorectal cancer effects by inducing apoptosis, cuproptosis and ferroptosis. Mitochondrial damage is a key marker of apoptosis, therefore, bioelectron microscopy of mitochondria was performed on the tumour tissues of Control and administered mice **(**Fig. 7C**)**. The mitochondria in the cRDT@FC group exhibited abnormal morphology, with the cristae disappearing. The Control group showed no abnormal manifestations. In summary, cRDT@FC can exert anti-colorectal cancer effects through various pathways, including the induction of apoptosis, cuproptosis, and ferroptosis, and has great potential in colorectal cancer treatment.

## 4 Conclusion

This study addresses the critical challenge of poor chemotherapy efficacy in colorectal cancer treatment, specifically the resistance to apoptosis. The current study successfully designed and constructed a multifunctional intelligent nanoparticle, cRDT@FC. This nanoparticle integrates the targeting ligand cRGD, the ferroptosis inducer Fc, the chemotherapy prodrug CPT, and a copper ion chelating polymer, thereby achieving tumour-specific targeted delivery, GSH depletion, and multi-modal induction of tumour cell death. *In vitro* experiments demonstrated that cRDT@FC exhibits excellent stability, GSH responsiveness, and controlled drug release capabilities, efficiently entering colorectal cancer cells and inducing ROS bursts and mitochondrial damage. *In vivo* studies revealed that cRDT@FC is significantly enriched in tumour tissues and exhibits a potent inhibitory effect on transplanted tumour models (with an inhibition rate of approximately 88 ± 6.9 %) while maintaining good biological safety. Mechanistically, various technologies, including RNA-seq, confirmed that cRDT@FC effectively induces apoptosis, ferroptosis, and cuproptosis in tumour cells. In conclusion, cRDT@FC enhances anti-cancer efficacy through a “multi-pathway synergistic killing” strategy, providing a highly efficient and safe new treatment option for colorectal cancer and demonstrating the significant application potential of nanomaterials in precise tumour treatment.

## CRediT authorship contribution statement

**Xuna Xue:** Writing – original draft. **Zhibo Zhang:** Writing – original draft. **Qiyu Zhang:** Methodology. **Xuerong Zhao:** Investigation. **Lina Xu:** Methodology. **Lianhong Yin:** Writing – review & editing. **Jinyong Peng:** Writing – review & editing. **Ning Wang:** Writing – review & editing.

## Declaration of competing interest

☑The authors declare that they have no known competing financial interests or personal relationships that could have appeared to influence the work reported in this paper.

## Data Availability

Data will be made available on request.
