# Supplementary material for: Copper/iron-based intelligent nanoparticles self-amplify apoptosis/ferroptosis/cuproptosis in colorectal cancer
Source: Mater Today Bio. 2025 Dec 23;36:102732. doi: 10.1016/j.mtbio.2025.102732 (PMC12818285; doi:10.1016/j.mtbio.2025.102732)
Supplement: Multimedia component 1 [file mmc1.docx]

**Copper/iron-Based Intelligent Nanoparticles Self-amplify Apoptosis/ferroptosis/cuproptosis in Colorectal Cancer**

Xuna Xue^1,#^, Zhibo Zhang^1,#^, Qiyu Zhang^1^, Xuerong Zhao^1^, Lina Xu^1^, Lianhong Yin^1,*^, Jinyong Peng^1,2*^, Ning Wang^1,^*

^1^ College of Pharmacy, Dalian Medical University, Western 9 Lvshunnan Road, Dalian 116044, China

^2^ College of Pharmacy, Hubei Shizhen Laboratory, Hubei University of Chinese Medicine, Wuhan 430065, China

*Corresponding author

Dr. Lianhong Yin

College of Pharmacy, Dalian Medical University, Dalian 116044, China

Email: yinlianhong1015@163.com

Dr. Jinyong Peng

College of Pharmacy, Dalian Medical University, Dalian 116044, China

College of Pharmacy, Hubei Shizhen Laboratory, Hubei University of Chinese Medicine, Wuhan 430065, China

Email: jinyongpeng2008@126.com

Dr. Ning Wang

College of Pharmacy, Dalian Medical University, Dalian 116044, China

Email: wangn06@dmu.edu.cn

^#^ These authors contribute equally: Xuna Xue and Zhibo Zhang

**Experimental section**

- **Chemicals and Materials**

Glutathione (GSH), camptothecin (CPT), N-(3-Dimethylaminopropyl)-N-ethylcarbodiimide (EDCI), 4-dimethylaminopyridine (DMAP), fluorescent dye DiR, 3-mercaptopropionic acid, cholesterol chloroformate, methoxy polyethylene glycols (PEG_2K_-OH, Mw = 2000 Da; (PEG_5K_-OH, Mw = 5000 Da), diethylenetriaminepentaacetic acid (DTPA), 2-hydroxyethyl disulfide, ferrocenecarboxylic acid, 2-hydroxyethyl disulfide and 1,2,4,5-cyclohexanetetracarboxylic acid dianhydride were purchased from Shanghai Macklin Biochemical Co., Ltd. Shanghai Yaxian Chemical Co., Ltd (China) provided peptides (cRGDyc). Adriamycin hydrochloride (DOX·HCl) was purchased from Shanghai Hao Yun Chemical Technology Co., Ltd. Oxygen Species Assay Kit (DCFH-DA) was purchased from Shanghai Beyotime Biotechnology Co., Ltd. BioTracker 575 Red Fe^2+^ Dye, purchased from Merck KGaA, Darmstadt, Germany. ALT, AST, Cr and Bun kits were purchased from Nanjing Jiancheng Bioengineering Research Institute Co., Ltd. Anti-GPX4, Caspase3, DLAT, SLCTA11, P53, BAX, FDX1, XCT, Integrin ɑⅤ, Integrin β3 and GAPDH antibodies were purchased from Proteintech Group, Inc. Precast PAGE Gel (4%-20%) were purchased from KeyGEN, Jiangsu, China. Tunel kit, ready-to-use DAPI staining solution, BCA Protein Assay Kit and Cell Counting kit-8 were purchased from Sevenbio, Beijing, China.

- **Synthesis of cRGD-PEG-TK-Chol**

From our previous work, Mal-PEG-TK-Chol were obtained [1]. The Mal-PEG-TK-Chol (270 mg, 0.1 mmol) alongside peptides (cRGDyc, 72 mg, 0.12 mmol) were dissolved in 10 mL anhydrous DMSO for 24 h. Thereafter, the reaction mixture was placed in a dialysis bag (with a molecular weight cut-off of 1000 Da) and dialyzed for 24 h. Subsequently, the solution was freeze-dried to acquire cRGD-PEG-TK-Chol (193.6 mg) with a yield of 58.6%, thereby confirming the product identity by ^1^H NMR.

- **Synthesis of Poly-SS-DOX**

A solution of 2-hydroxyethyl disulfide (2 mmol, 308 mg) and 1,2,4,5-cyclohexanetetracarboxylic acid dianhydride (2.1 mmol, 470.8 mg) was prepared in 10 mL of anhydrous DMF. Following magnetic stirring at 50 ℃ for a duration of 24 h, mPEG_5K_-OH (1000 mg) was introduced into the reaction mixture, which continued to react for an additional 24 h. The resultant mixture was then transferred into a dialysis bag (with a molecular weight cut-off of 8000 Da) and dialyzed over 48 h. After this period, the solution was subjected to freeze-drying under reduced pressure to yield Poly-SS-COOH, which was subsequently analyzed using ^1^H NMR.

Poly-SS-COOH (1000 mg) was dissolved in DMSO (20 mL). The solution of EDCI (230 mg, 1.2 mmol) and DMAP (146 mg, 1.2 mmol) was added to the above-mentioned solution, and the mixture was stirred at room temperature for 2 h. Next, the solution of DOX·HCl (696 mg, 1.2 mmol) in 20 mL of DMSO was added, and the reaction was stirred for 24 h at room temperature. Subsequently, the product was purified by dialyzing in a membrane (with a molecular weight cut-off of 8000 Da) against deionized for 48 h and filtered, lyophilized to obtain the red product Poly-SS-DOX. The successful synthesis of Poly-SS-DOX was confirmed by ^1^H-NMR.

- **Synthesis of PEG-SS-CPT**

In a 100 mL single-mouthed flask, combine mPEG_2K_-OH (2 g, 1 mmol), succinic anhydride (0.5 g, 5 mmol), and DMAP (0.06 g, 0.5 mmol). Add 100 mL of anhydrous DCM and allow the reaction to proceed at room temperature for 24 h. Upon completion of the reaction, remove the solvent using rotary evaporation. Subsequently, add 20 mL of saturated brine and filter to eliminate the white precipitate. Following this, perform three extractions with DCM, combine the organic phases, dry with anhydrous Na_2_SO_4_, filter, and concentrate under reduced pressure. A total of 1.53 g of white solid PEG_2K_-COOH was obtained, resulting in a yield of 72.8%.

From our previous work, CPT-SS-OH were obtained [2]. PEG_2K_-COOH (1 g, 0.5 mmol) was dissolved in DCM (20 mL). The solution of EDCI (118 mg, 0.6 mmol) and DMAP (7.3 mg, 0.06 mmol) was added to the above-mentioned solution, and the mixture was stirred at room temperature for 2 h. Subsequently, we introduced a solution of CPT-SS-OH (317 mg, 0.6 mmol) in 50 mL of anhydrous DCM into the reaction mixture and stirred at room temperature for 24 h. Then, the mixture was rinsed with 0.1 M HCl, brine, and water. This was followed by separating the organic layer, drying over anhydrous Na_2_SO_4_, filtration, solvent removal in a vacuum, and crude product purification using silica gel chromatography (CH_2_Cl_2_/CH_3_OH: 20:1). This was conducted to obtain PEG-SS-CPT as a yellow solid (689 mg, yield 54.8%), thereby confirming product identity by ^1^H NMR.

- **Synthesis of PEG-DTPA-Fc**

The synthesis of PEG-DTPA-Fc was reported by the previous work of this group [3].

- **Characterization of cRDT@FC**

The actual drug loadings of Poly-SS-DOX and PEG-SS-CPT were quantified using UV-visible spectrophotometry (UV/Vis). Additionally, the size distribution and morphology of cRDT@FC were characterized through dynamic light scattering (DLS, Malvern Zetasizer Nano ZS) and transmission electron microscopy (TEM, JEM-2000EX*, Japan). JEM-F200

- **Cumulative release of CPT in cRDT@FC**

Dialysis bags containing cRDT@FC were placed in 100 mL of PBS at pH 7.4, 5.5 and in PBS with 5 mM GSH respectively. At predetermined time intervals, 2 mL of the sample solution was with draw and replaced with an equal volume of the corresponding medium. The concentrations of CPT in the withdraw sample solution were measured using a fluorescence spectrometer.

- **Cellular uptake**

Cellular uptake in vitro was investigated using inverted fluorescence microscopy. CT26 cells were inoculated in 6-well plates at a specific density and cultured overnight in a 5 % CO_2_ atmosphere at 37 ℃. After removing the culture medium, fresh culture medium containing cRDT@FC and DT@FC was added, and incubation continued for 0, 1, 2, 3, 4, and 5 hours. Finally, the culture medium was discarded, and the cells were washed three times with cold PBS. The nuclei of the cells were then stained with DAPI for 10 minutes and observed using inverted fluorescence microscopy.

- **Cytotoxicity study *in vitro***

The cytotoxic effects of cRDT@FC on tumour cells (CT26, MC38) and normal cells (Nrk-49F) were assessed using the CCK-8 assay. Tumour cells were inoculated into 96-well plates at a specific density and cultured overnight in a 5% CO_2_ atmosphere at 37 ℃. Subsequently, CPT, cRDT, DT@FC and cRDT@FC were added in a concentration gradient of 0, 0.5, 1, 1.5 and 2 μg/mL based on CPT, while the Control group received serum-free culture medium. After 24 hours of treatment, 10 μL of CCK-8 reagent was added to each well and incubated for 1 hour. The absorbance at 450 nm was then measured. Fo normal cells (Nrk-49F), only CRDT@FC was administered at varying CPT concentrations.

- **Iron ion fluorescence assay**

BioTracker^TM^ 575 Red Fe^2+^ Dye was utilized to detect Fe^2+^ levels in vitro. CT26 cells were inoculated in 6-well plates at a specific density and cultured overnight in a 5% CO_2_ atmosphere at 37 °C. Various concentrations of PEG-DTPA-FC (n=3) were added and incubated for 4 hours. The cells were subsequently stained using Bio Tracker 575 Red Fe^2+^ Dye Kit and observed under an inverted fluorescence microscope.

- **Cellular ROS level detection assay**

CT26 cells were inoculated into 6-well plates at a specific density and cultured overnight at 5 % CO_2_ and 37 °C. The cells were divided into five groups (n=3): Control, CPT, cRDT, DT@FC and cRDT@FC. After removing the culture medium, add various preparations according to the groups and incubated for 4 hours (CPT at a concentration of 2 μg/mL). The The cells were then stained using the ROS Assay Kit and observed with an inverted fluorescence microscope.

- **Bio-electron microscopy**

CT26 cells were inoculated at a specific density in 150 mm cell culture dishes and incubated overnight at 5% CO_2_ and 37 ℃. The cells were divided into two groups (n=3): the Control group and the cRDT@FC group. The cRDT@FC group received cRDT@FC at a CPT concentration of 2 μg/mL and was incubated for 24 hours. The Control group was supplemented with serum-free culture medium. After 24 hours, the cells were collected, fixed with electron microscope fixative, and observed using a bio-electron microscope.

- ***In vivo* acute toxicity experiments**

Thirty male Balb/c mice were randomly divided into five groups: a Control group and four cRDT@FC groups (CPT: 2 mg/kg, 4 mg/kg, 6 mg/kg, 8 mg/kg). Except for the Control group, the mice in the other four groups were injected intravenously with the specified dose of cRDT@FC via the tail vein. Twenty-four hours later, the mice were anesthetized with isoflurane, and cardiac function was assessed using small animal ultrasound and electrocardiogram. The values for ejection fraction (EF%), fractional shortening (FS%), left ventricular diastolic diameter (LVID d), left ventricular systolic diameter (LVID s), left ventricular end-diastolic volume (LVED V) and left ventricular end-systolic volume (LVES V), and the E/A ratio were calculated for all mice. Subsequently, the mice were subsequently euthanized, and whole blood along with major organs were collected for biochemical analysis and H&E staining

- **Fluorescence imaging studies *in vivo***

cRDT@DIR, DT@DIR, and DIR were administered to tumour-bearing mice via the tail vein, followed by *in vivo* imaging at various time points. Twenty-four hours later, the mice were euthanized, and the tumours along with major organs (heart, liver, spleen, lungs, and kidneys) were harvested, rinsed with cold saline, and subjected to fluorescence imaging to assess the biological distribution of cRDT@DIR, DT@DIR, and DIR.

- ***In vivo* anti-tumour activity and biosafety evaluation**

CT26 cells (2×10^7^) were inoculated into the left subcutaneous side of male Balb/c mice. when the tumour volume reached 50 mm^3^, the tumour-bearing mice were randomly divided into five groups (n=5), and injected into the tail vein with PBS, CPT (6 mg/kg), cRDT (CPT: 6 mg/kg), DT@FC (CPT: 6 mg/kg) and cRDT@FC (CPT: 6 mg/kg) respectively. Mice were injected into the tail vein every two days for a total of five injections, and the tumour volume and body weight were measured and recorded throughout the experiment. Tumour volume was calculated using the formula: V=L×W^2^/2, where L is the longest dimension of the tumour and W is the shortest dimension. After 10 days of treatment, the mice were anesthetized with isoflurane, and cardiac function was assessed using small animal ultrasound and electrocardiogram. The numerical values of EF%, FS%, LVID d, LVID s, LVED V, LVES V and E/A were calculated for all mice. Subsequently, the mice were anesthetized, and tumour tissues were collected and stained with Tunnel, BAX, FDX1, and XCT. Tumour tissues from the Control group and the cRDT@FC group were fixed with electron microscope fixative and examined by Bio-TEM. Whole blood and major organs were collected for biochemical analysis and H&E staining.

- **Western blot analysis**

CT26 cells were inoculated at a specific density in 150 mm cell culture dishes and cultured overnight in a 5% CO_2_ atmosphere at 37 °C. The cells were divided into five groups (n=3): Control, CPT, cRDT, DT@FC and cRDT@FC. After removing the culture medium, an equal amount of CPT concentration (2 μg/mL) of CPT, cRDT, DT@FC and cRDT@FC was added and incubated overnight. Proteins from the cells were extracted using RIPA buffer and quantified with BCA kit. Protein lysates (40-50 μg) were separated by PAGE@Gel. Membranes were incubated with primary antibodies against BAX, GPX4, SLC7A11, DLAT, P53, FDX1 and XCT, followed by incubation with appropriate goat anti-rabbit/mouse Poly-HRP secondary antibodies. All blots were analyzed using Image J software, with β-actin serving as an internal reference.

CT26, MC38, Nrk-49F, and Huvec cells were inoculated at a specific density in 150-mm cell culture dishes and incubated overnight in a 5% CO_2_ atmosphere at 37 °C. After 24 hours, cells were collected, and total proteins were extracted and quantified using a BCA kit. Protein lysates (40-50 μg) were separated by PAGE@Gel. Membranes were incubated with primary antibodies against Integrin ɑ V and Integrin β 3, followed by incubation with appropriate goat anti-rabbit/mouse Poly-HRP secondary antibodies. All blots were analyzed using Image J software, with β-actin serving as an internal reference.

- **Hemolysis test**

Blood from male Balb/c mice was diluted 30-fold with saline and added to 24-well plates in a volume of 1 mL per well. Different concentrations of cRGD-PEG-TK-Chol, Poly-SS-DOX, PEG-SS-CPT and PEG-DTPA-Fc solutions (100, 200, 300, and 400 μg/mL) were added and incubated at 37 °C for 4 hours. After incubation, 0.9 mL of the blood solution was transferred to a centrifuge tube and centrifuged at 2000 rpm for 5 minutes to remove intact red blood cells. Subsequently, 200 μL of the supernatant was transferred to a 96-well plate, and the absorbance at 540 nm was measured using an enzyme marker. Erythrocytes treated with 1% Triton X-100 served as a positive control.

- **mRNA-sequencing analysis**

Total RNA was extracted from CT26 cells in the control group (n = 3) and the cRDT@FC group (n = 3) using TRIzol (Invitrogen, USA) according to the manufacturer's instructions. RNA purification, reverse transcription, library construction, and sequencing were conducted at Shanghai Majorbio Bio-Pharm Technology Co. (China). the concentration of CPT in the cRDT@FC group was 2 μg/mL.

**References**

[1] M. Gao, L. Yin, B. Zhang, Z. Dong, W. Jiang, Z. Bai, X. Zhao, L. Xu, N. Wang, J. Peng, Targeting Ischemic Myocardium: Nanoparticles Loaded with Long Noncoding RNA AK156373 siRNA Alleviate Myocardial Infarction, ACS Nano 19(19) (2025) 18475-18491. https://doi.org/10.1021/acsnano.5c01641.

[2] N. Wang, Y. Liu, D. Peng, Q. Zhang, Z. Zhang, L. Xu, L. Yin, X. Zhao, Z. Lu, J. Peng, Copper‐Based Composites Nanoparticles Improve Triple‐Negative Breast Cancer Treatment with Induction of Apoptosis‐Cuproptosis and Immune Activation, Advanced Healthcare Materials 13(28) (2024). https://doi.org/10.1002/adhm.202401646.

[3] D. Peng, Z. Zhang, Q. Zhang, Y. Liu, N. Wang, Organic molecule-based nanoparticles for multimodal colorectal cancer therapy: Oxidative stress-induced apoptosis and ferroptosis, Chemical Engineering Journal 511 (2025). https://doi.org/10.1016/j.cej.2025.162259.

**Table. S1** Summary Table of Nanoparticle Composition

| NPs | Components and proportions | Mass ratio |
| --- | --- | --- |
| DT@FC  cRDT  cRDT@FC | Poly-SS-DOX/PEG-SS-CPT/PEG-DTPA-Fc/CuCl_2_  cRGD-PEG-TK-Chol/Poly-SS-DOX/PEG-SS-CPT  cRGD-PEG-TK-Chol/Poly-SS-DOX/PEG-SS-CPT/PEG-DTPA-Fc/CuCl_2_ | 2.2:2:4:4  1:2.2:2  1:2.2:2:4:4 |
| DT@DIR | Poly-SS-DOX/PEG-SS-CPT/DIR | 2.2:2:1 |
| cRDT@DIR | cRGD-PEG-TK-Chol/Poly-SS-DOX/PEG-SS-CPT/DIR | 1:2.2:2:1 |

**Fig. S1** Synthetic route of cRGD-PEG-TK-Chol.

**Fig. S2** Synthetic route of Poly-SS-DOX.

**Fig. S3** Synthetic route of PEG-SS-CPT.

**Fig. S4** Synthetic route of PEG-DTPA-Fc.


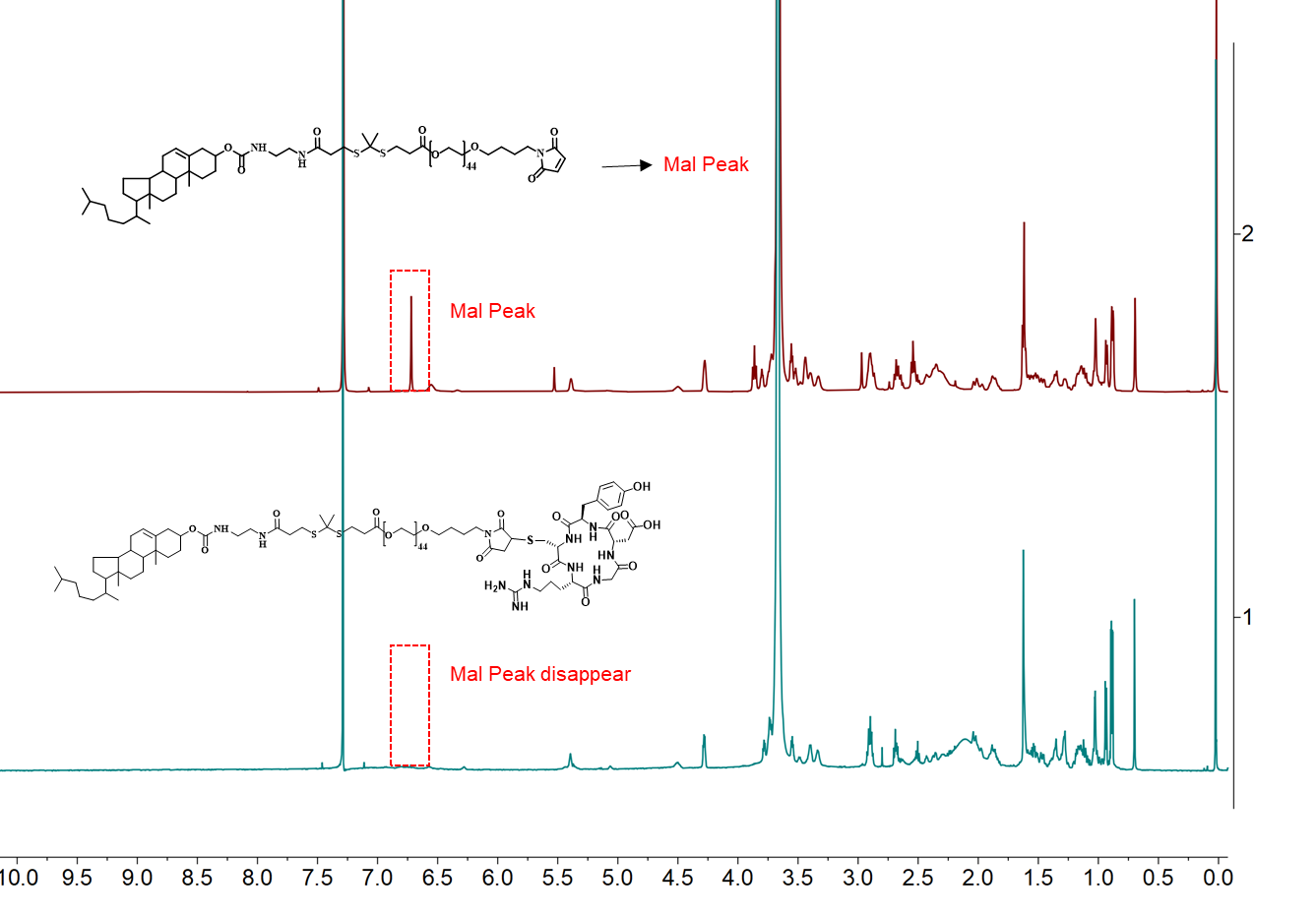


**Fig. S5** ^1^H-NMR spectra of Mal-PEG-TK-Chol and cRGD-PEG-TK-Chol.


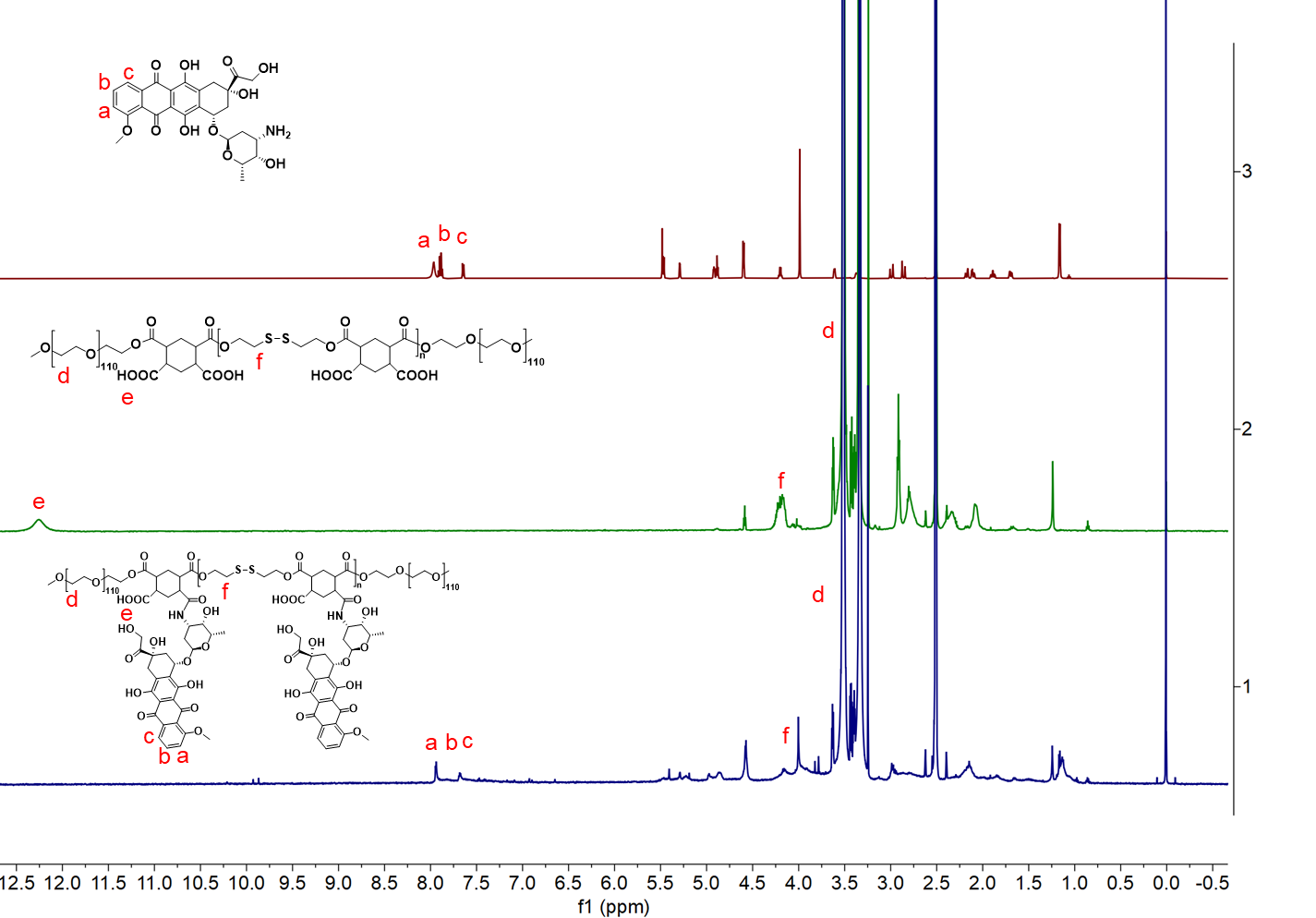


**Fig. S6** ^1^H-NMR spectra of DOX, Poly-SS-COOH and Poly-SS-DOX.


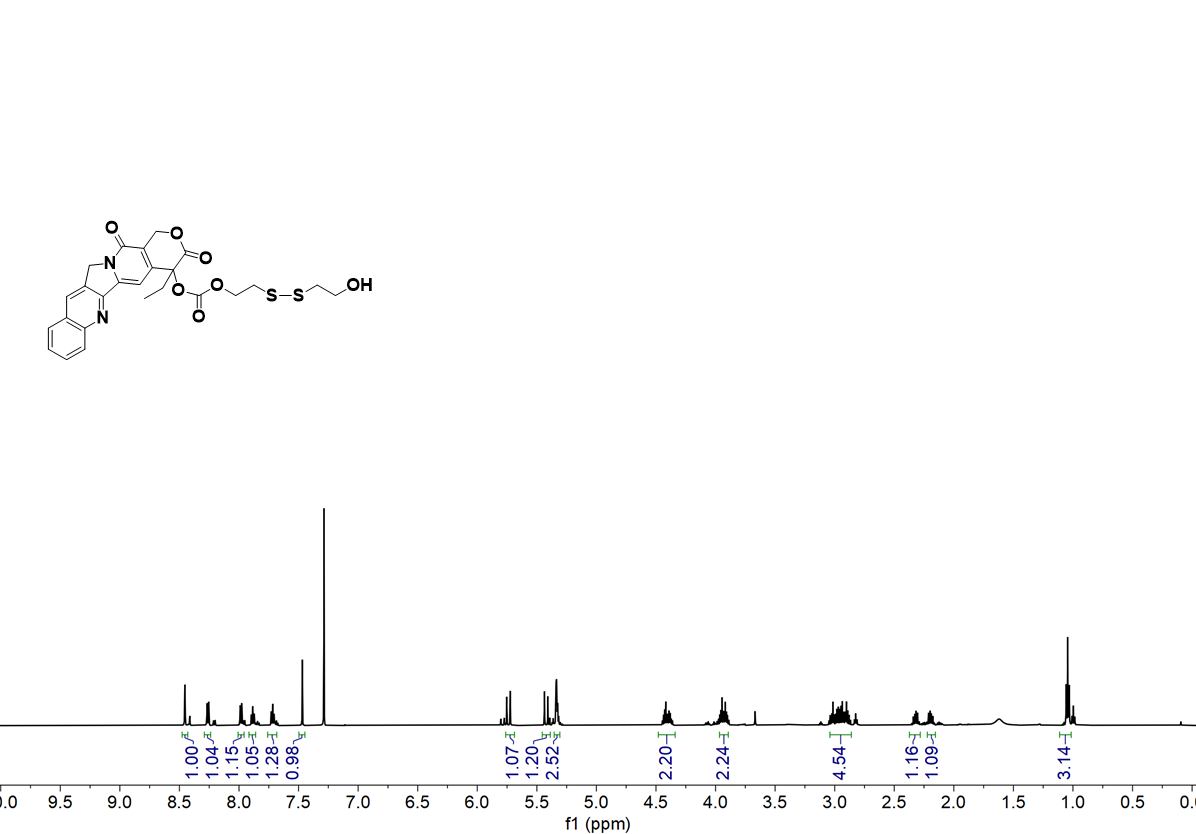


**Fig. S7** ^1^H-NMR spectra of CPT-SS-OH.


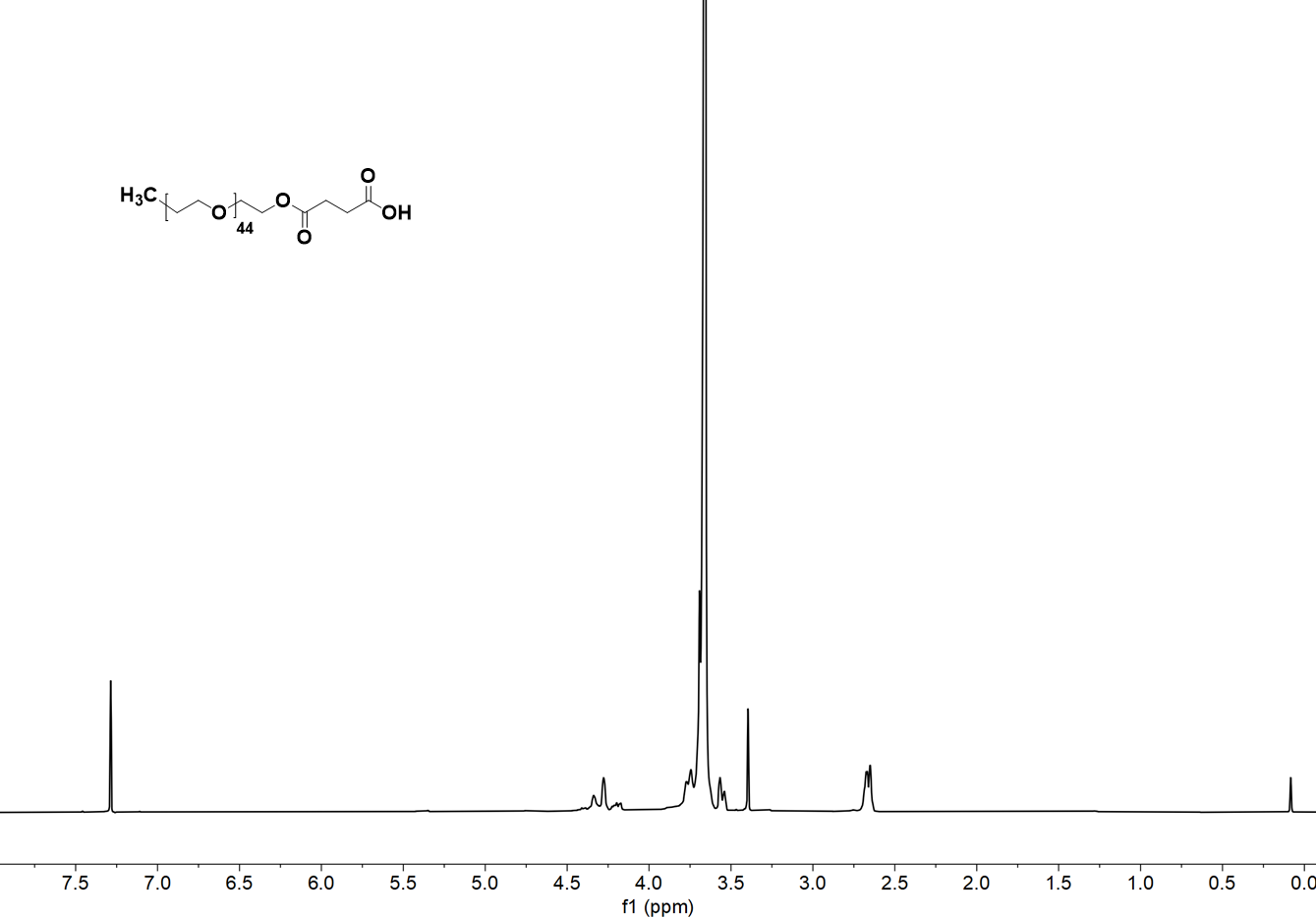


**Fig. S8** ^1^H-NMR spectra of PEG_2K_-COOH.


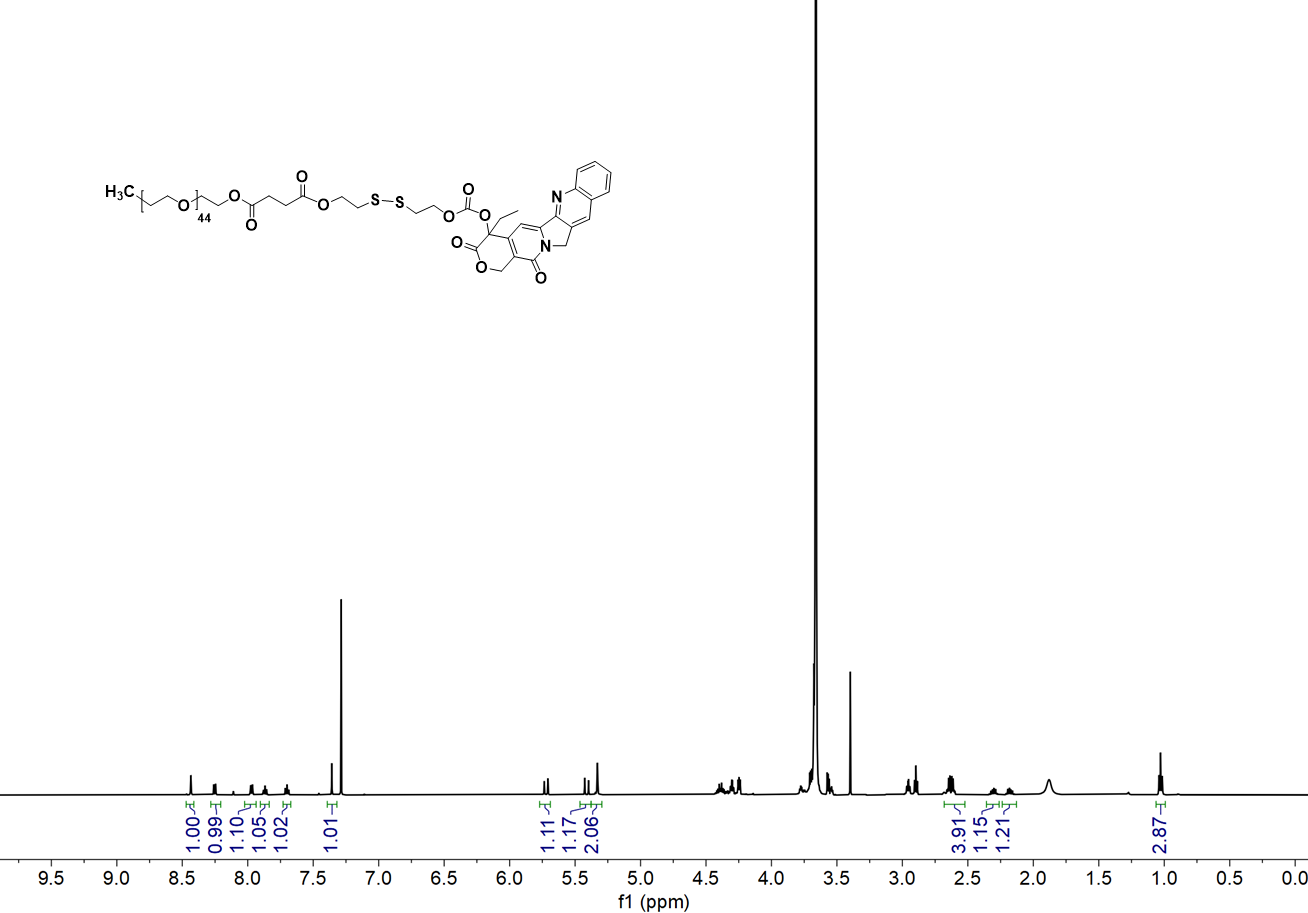


**Fig. S9** ^1^H-NMR spectra of PEG-SS-CPT.


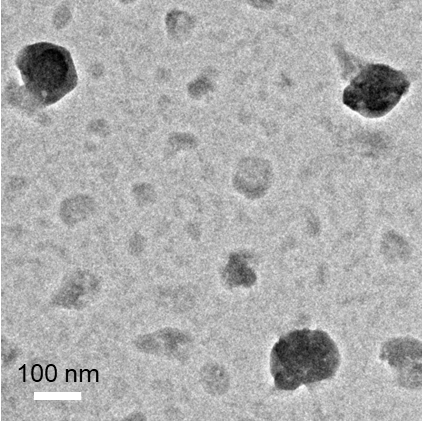


**Fig. S10** TEM diagram of cRDT@FC.


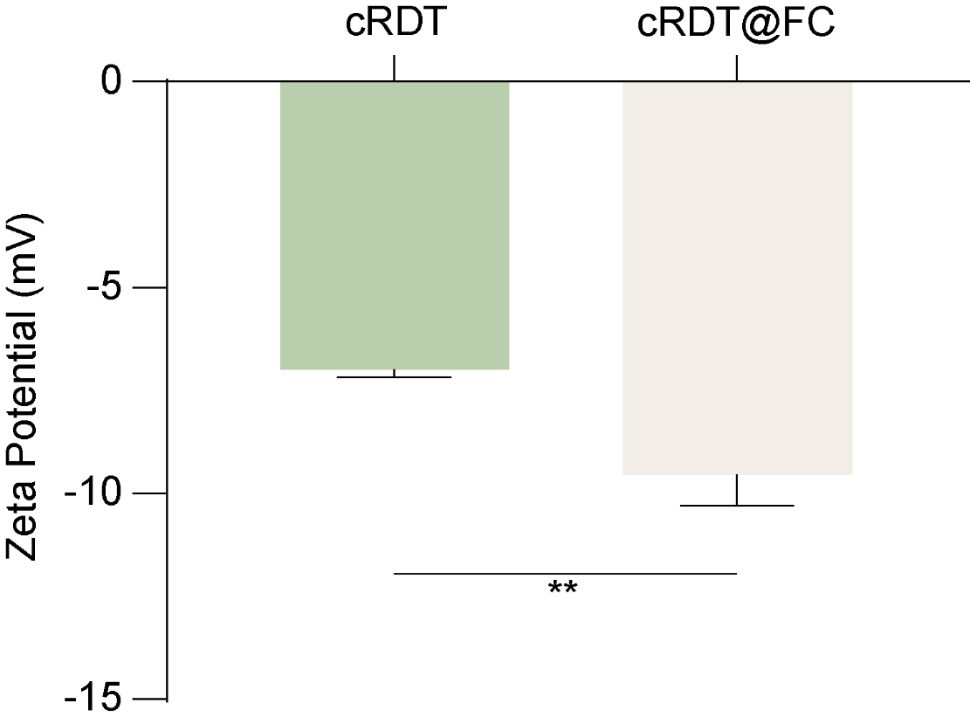


**Fig. S11** Zeta Potential of cRDT@FC. **p＜0.01.


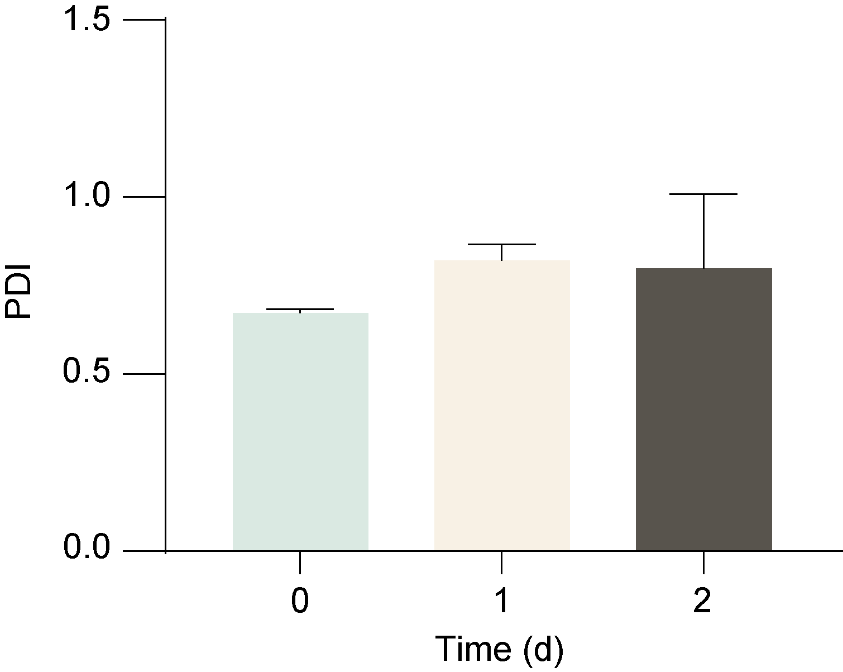


**Fig. S12.** PDI of cRDT@FC in 10% FBS conditions with 2 days.


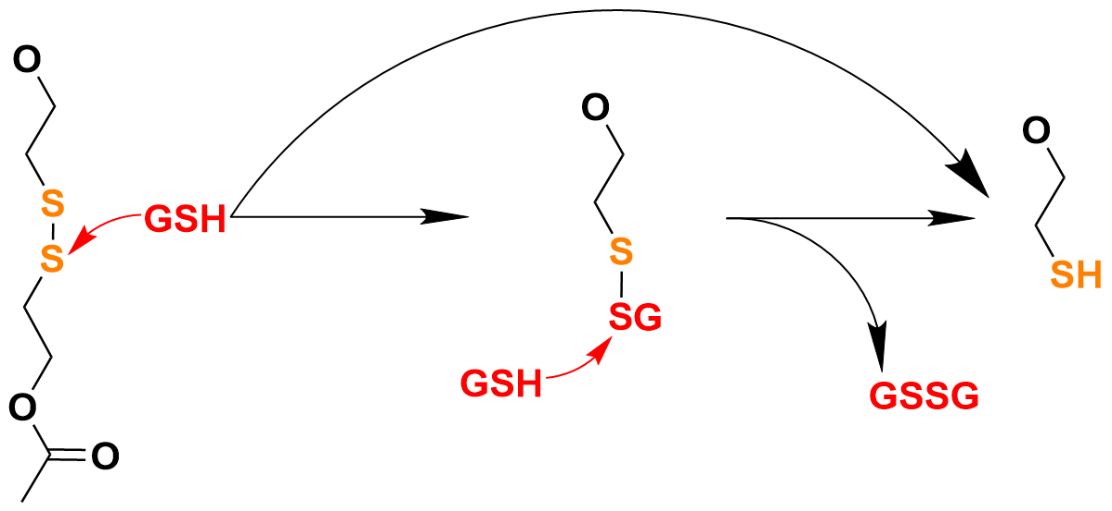


**Fig. S13.** The mechanism diagram of disulfide bond consuming GSH.
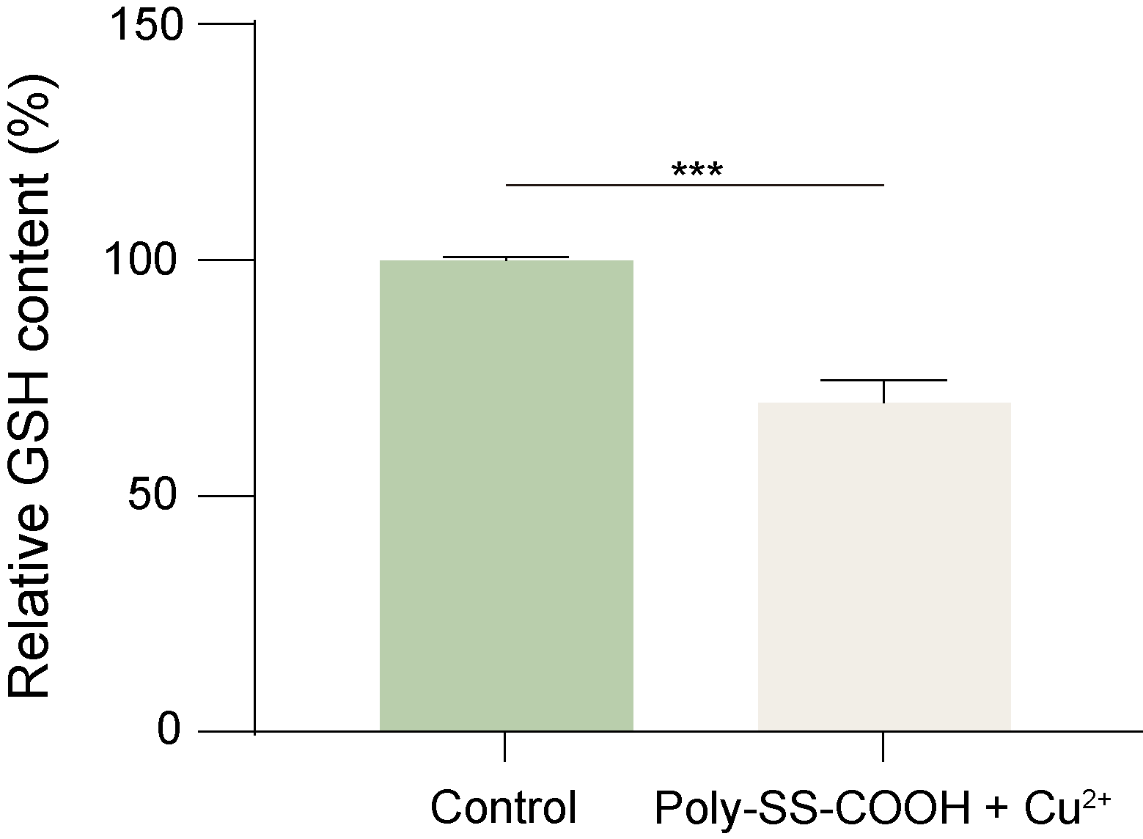


**Fig. S14.** Relative GSH content in CT26 cells after treatment with 50 μg/mL Poly-SS-COOH and 5 μg/mL Cu^2+^. ***p＜0.001.


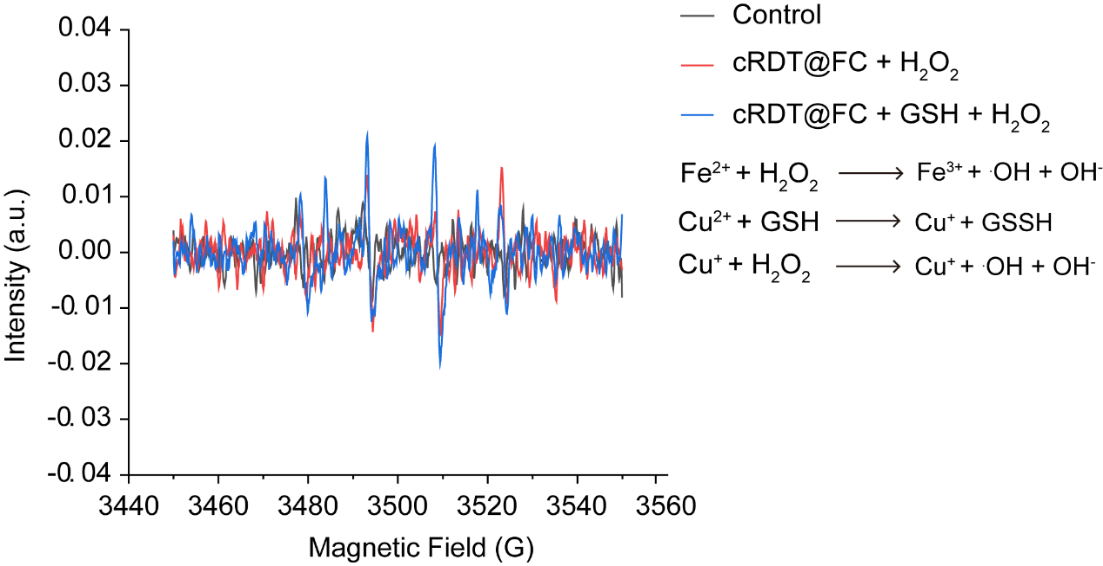


**Fig. S15.** ESR spectra of PBS and cRDT@FC with TEMP as the spin trap.


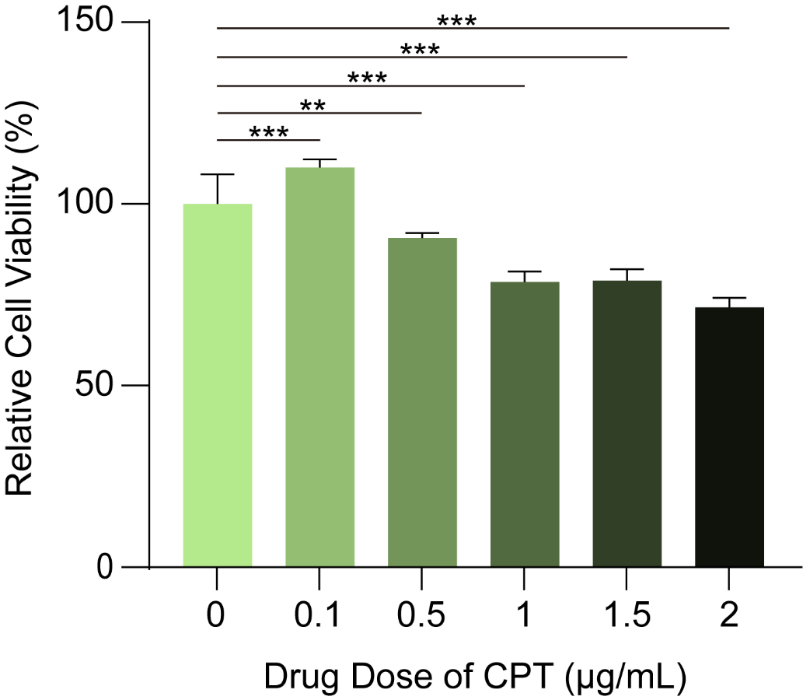


**Fig. S16.** The cell viability of different preparations to Nrk-49F cells after 24 h (n=6). **p＜0.01, ***p＜0.001.


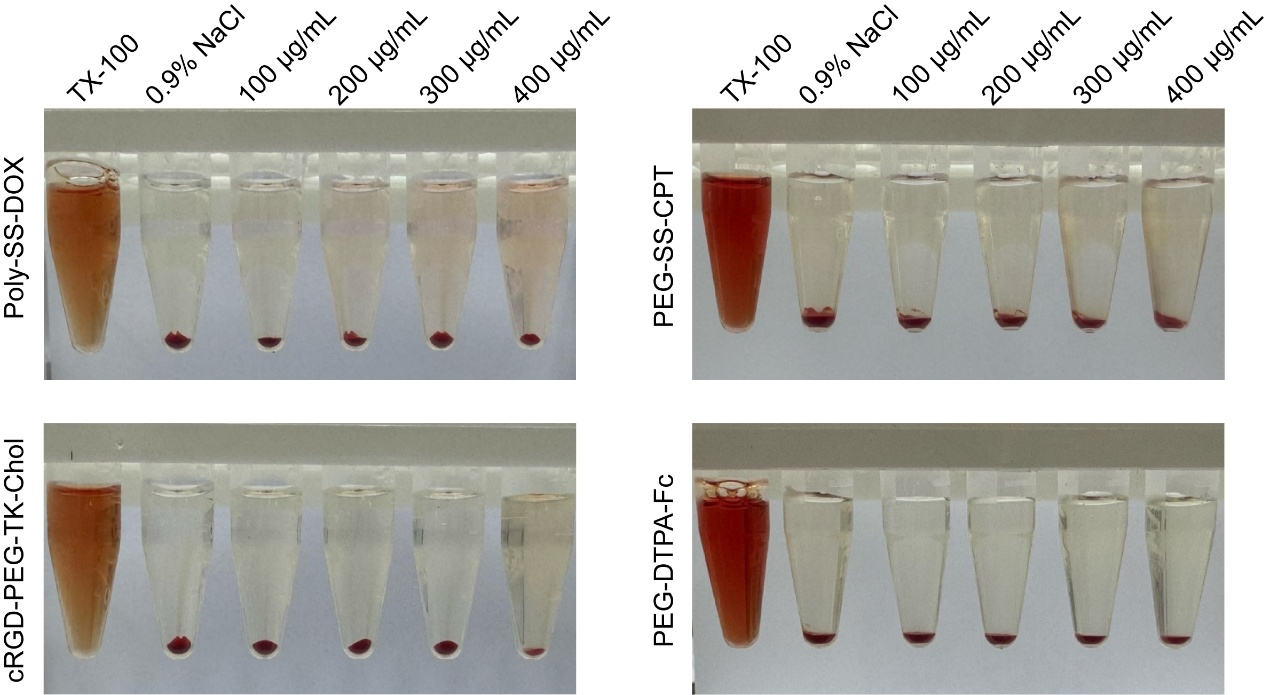


**Fig. S17** Hemolysis picture of Poly-SS-DOX, cRGD-PEG-TK-Chol, PEG-SS-CPT, PEG-DTPA-Fc at different concentration.


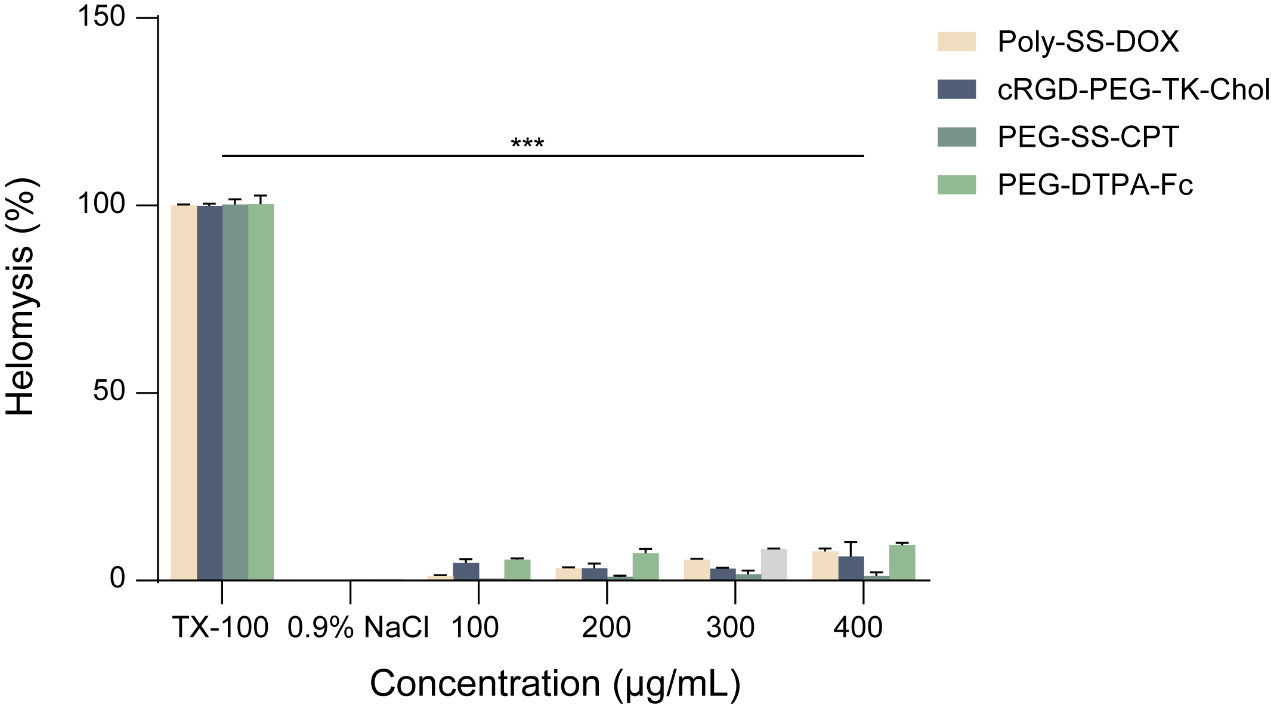


**Fig. S18** Hemolysis rate of Poly-SS-DOX, cRGD-PEG-TK-Chol, PEG-SS-CPT and PEG-DTPA-Fc. Date are presented as mean ± SD (n=3). Compared to the TX-100,***p＜0.001.


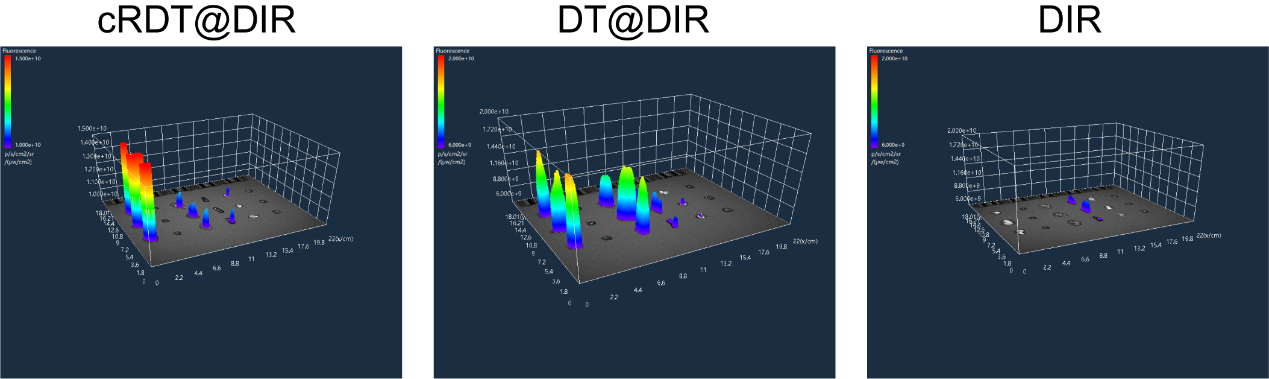


**Fig. S19** Fluorescent 3D images of Figure 4B.


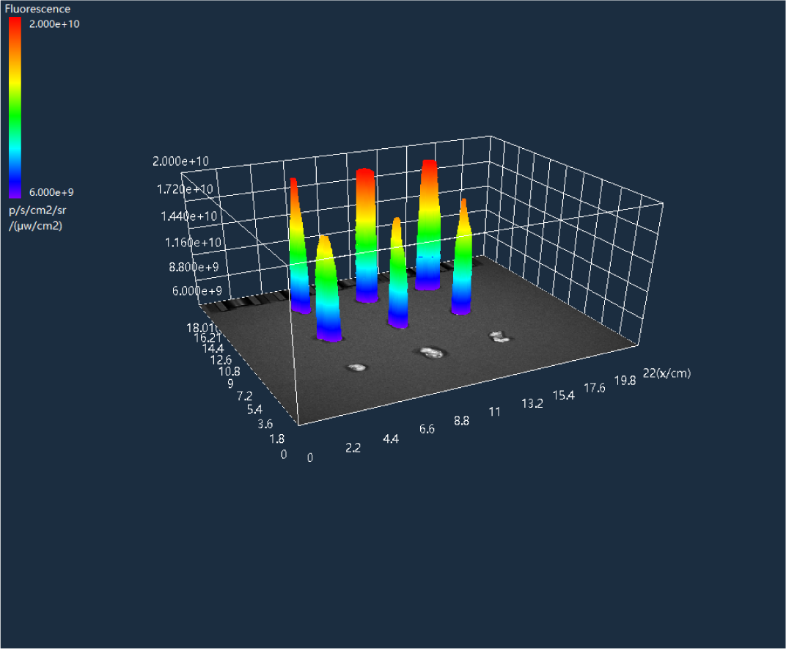


**Fig. S20** Fluorescent 3D images of Figure 4C


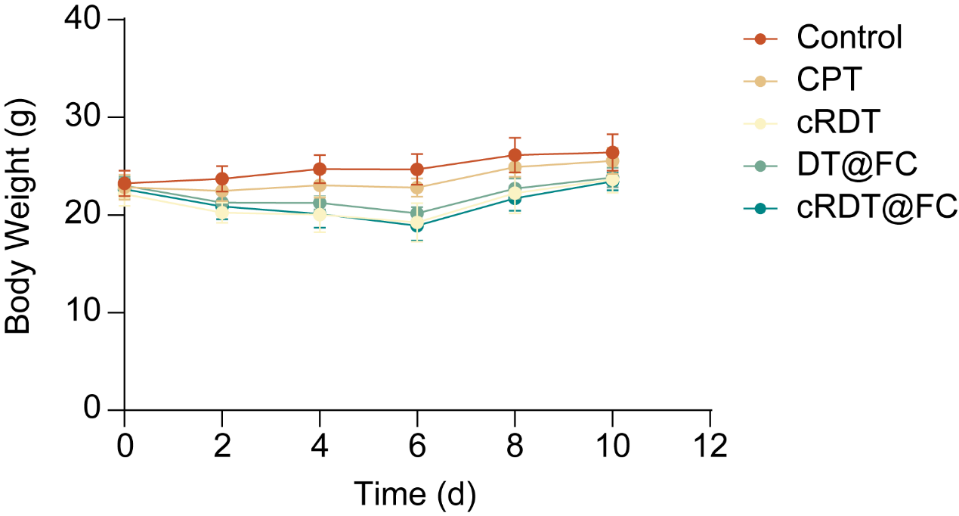


**Fig. S21** Body Weight change curves of mice for every other treatment group during the treatment period.


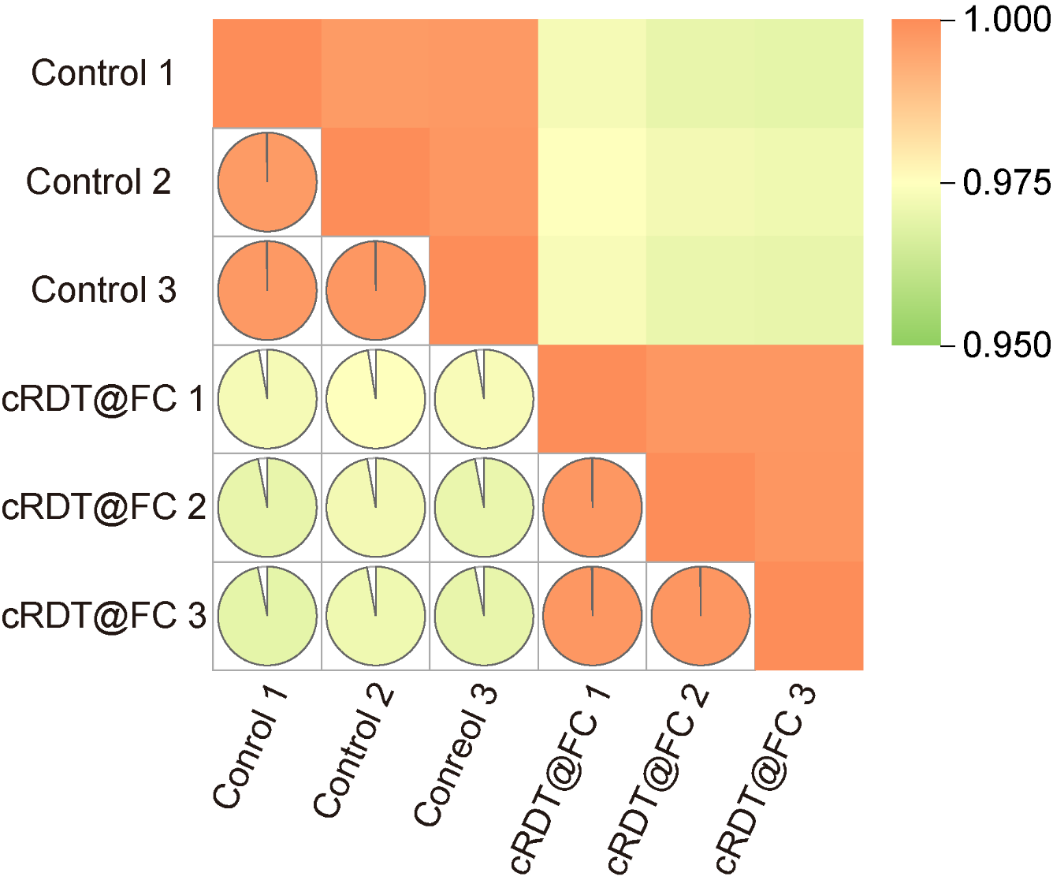


**Fig. S22** Correlation analysis of the RNA-seq data of allografts under cRDT@FC group and Control group.


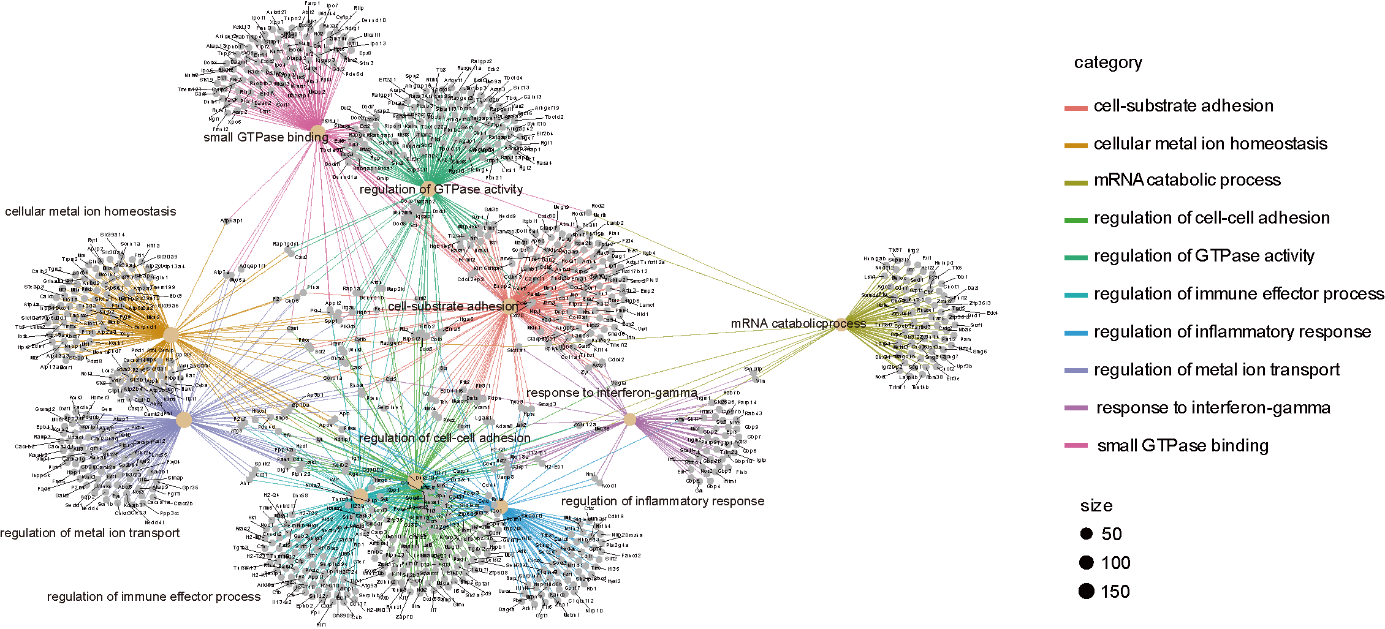
 **Fig. S23** Enrichment analysis of Up and Down genes of DEGs by GO pathway.


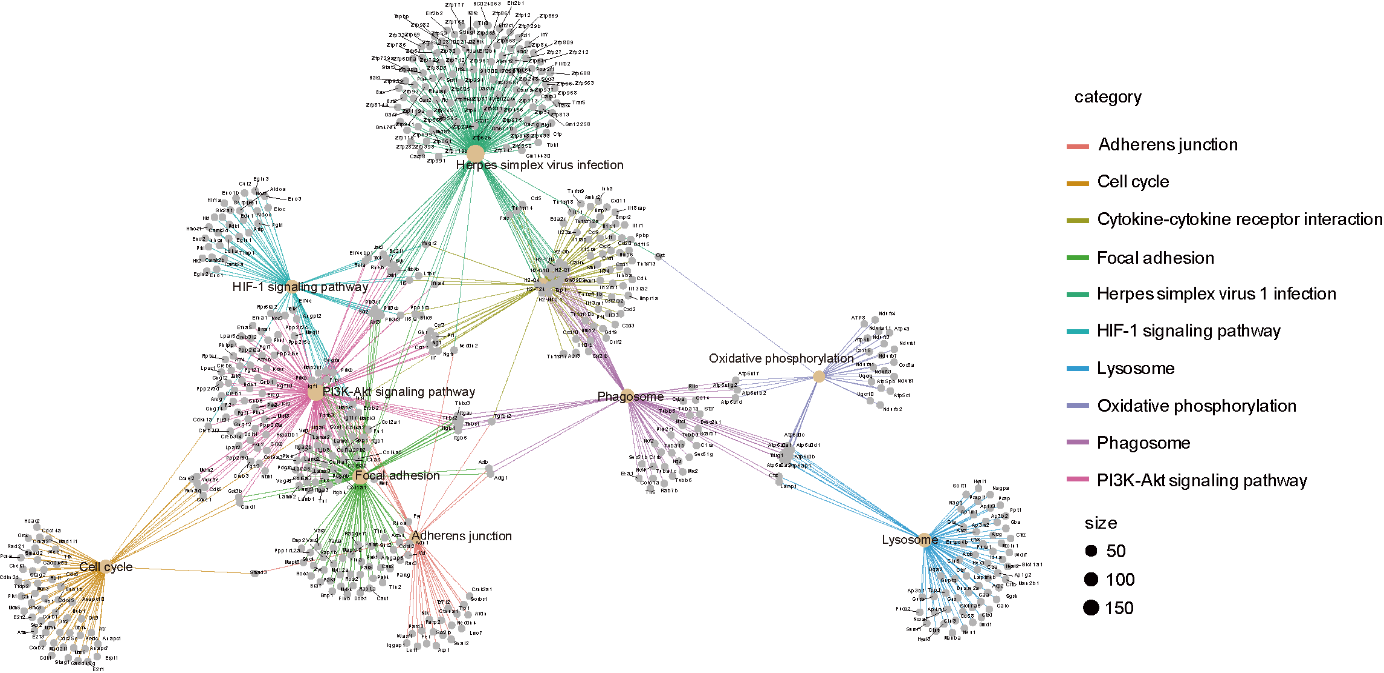
 **Fig. S24** Enrichment analysis of Up and Down genes of DEGs by KEGG pathway.


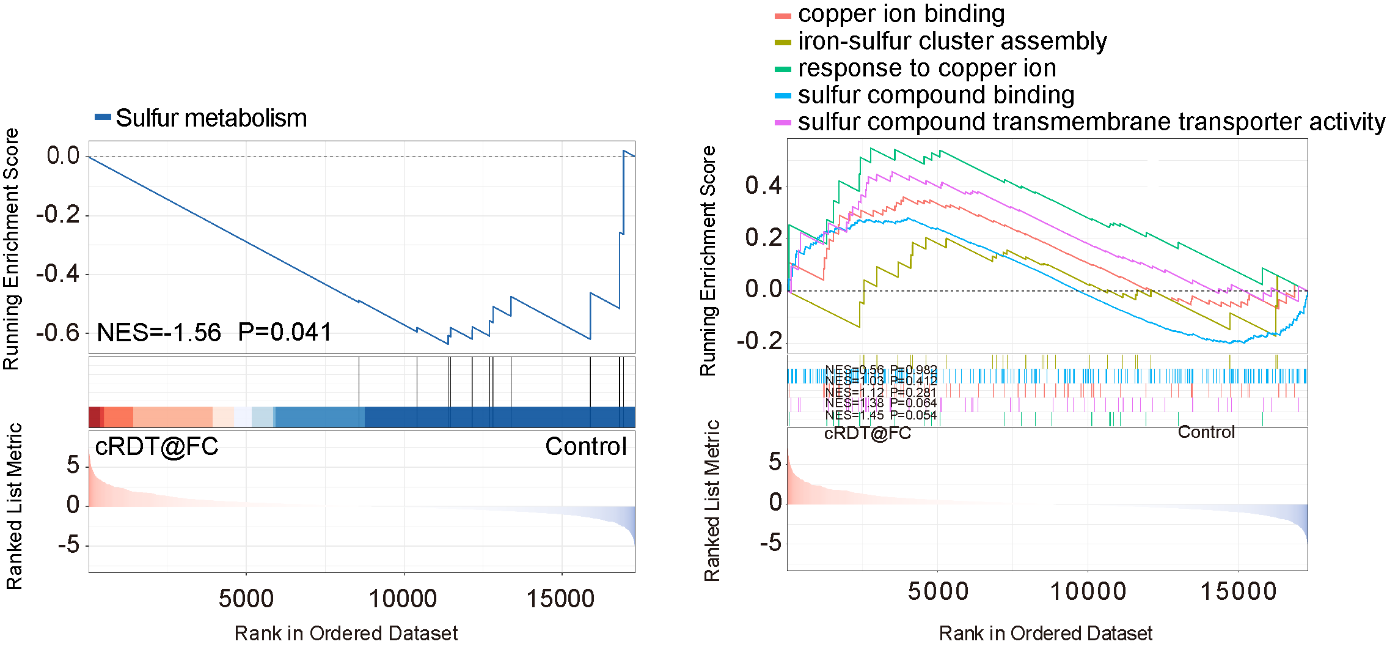
 **Fig. S25** GSEA of Cuproptosis related pathways in cRDT@FC treated CT26 cells versus Control.
